# Approximating the global economic (market) value of farmed animals

**DOI:** 10.1016/j.gfs.2023.100722

**Published:** 2023-12

**Authors:** Peggy Schrobback, Gabriel Dennis, Yin Li, Dianne Mayberry, Alexandra Shaw, Theodore Knight-Jones, Thomas Lloyd Marsh, Dustin L. Pendell, Paul R. Torgerson, William Gilbert, Benjamin Huntington, Kassy Raymond, Deborah A. Stacey, Theresa Bernardo, Mieghan Bruce, K. Marie McIntyre, Jonathan Rushton, Mario Herrero

**Affiliations:** a Global Burden of Animal Diseases Programme, UK^1^; b Commonwealth Scientific and Industrial Research Organisation (CSIRO), Agriculture & Food, St Lucia, Australia; c Murdoch University, Murdoch Veterinary School, Perth, Australia; d Infection Medicine, Deanery of Biomedical Sciences, Edinburgh Medical School, The University of Edinburgh, UK; e International Livestock Research Institute (ILRI), Addis Ababa, Ethiopia; f Washington State University, School of Economic Science, Pullman, USA; g Kansas State University, Department of Agricultural Economics, Manhattan, USA; h University of Zurich, Vetsuisse Faculty, Section of Epidemiology, Zurich, Switzerland; i University of Liverpool Institute of Infection, Veterinary and Ecological Sciences, Liverpool, UK; j University of Guelph, School of Computer Science, Guelph, Canada; k University of Guelph, Ontario Veterinary College, Department of Population Medicine, Guelph, Canada; l Murdoch University, School of Veterinary Medicine and Centre for Biosecurity and One Health, Harry Butler Institute, Perth, Australia; m Newcastle University, Evidence and Policy Group, School of Natural and Environmental Sciences, Newcastle, UK; n Cornell University, Department of Global Development, College of Agriculture and Life Sciences, and Cornell Atkinson Center for Sustainability, Ithaca, USA

**Keywords:** Livestock, Aquaculture, Farmed animals, Total economic value, Market, Food security, Non-market value

## Abstract

Understanding the global economic importance of farmed animals to society is essential as a baseline for decision making about future food systems. We estimated the annual global economic (market) value of live animals and primary production outputs, e.g., meat, eggs, milk, from terrestrial and aquatic farmed animal systems. The results suggest that the total global market value of farmed animals ranges between 1.61 and 3.3 trillion USD (2018) and is expected to be similar in absolute terms to the market value of crop outputs (2.57 trillion USD). The cattle sector dominates the market value of farmed animals. The study highlights the need to consider other values of farmed animals to society, e.g., finance/insurance value and cultural value, in decisions about the sector’s future.

## Highlights

• Global market value of farmed animals lays within 1.61–3.3 trillion USD (2018).
• Market value of animals is likely similar to global crop output value (2.57 trillion USD).
• Cattle contribute largest proportion to global value (and mass) of farmed animals.
• Non-market values of farmed animals need to be considered in global decision making.

## 1 Introduction

Terrestrial and aquatic farmed animals are vital for global society, providing food and nutrition, and other benefits such as draught power and financial services (e.g., cashable livestock assets, livestock assets as credit/collateral in the absence of formal financial services) and cultural value (Gandini and Villa, 2003; Herrero et al., 2013; Herrero et al., 2009; Moll, 2005; J. Smith et al., 2013; Thornton, 2010; Zane and Pica-Ciamarra, 2021). An understanding of the economic importance of farmed animals to society is essential as a baseline for decision making about the future of the global food system.

Previous studies have demonstrated the global importance of the farmed animal sector and challenges within the livestock food system from different perspectives. For example, emphasis was put on animal protein and its important roles in global food and nutrition security, and poverty alleviation (Enahoro et al., 2018; Henchion et al., 2017; Thornton et al., 2002). Other studies have assessed the environmental impact of farmed animals through evaluations of global livestock biomass, resource use (e.g., land, water) and negative externalities such as greenhouse gas emissions, loss of biodiversity and impact on other ecosystem services (Caro et al., 2014; Gerber et al., 2013; Heinke et al., 2020; Pelletier and Tyedmers, 2010; Steinfeld et al., 2006). Further, the role of terrestrial livestock in developing countries has also been explored (Herrero et al., 2013; Thornton, van de Steeg, Notenbaert and Herrero, 2009). The global status of aquatic farmed animal production has historically been assessed separately from livestock sector development (Naylor et al., 2000, 2021; Tacon, 2020) despite similar risks (e.g., environmental impacts) and benefits (e.g., provision of food, nutrition).

Missing from the existing inventory is an assessment of the economic value that terrestrial livestock and aquatic farmed species contribute to global society. Such information is critical to guide future global and national investments and policy formulation on aspects such as production system development, animal health and welfare, environmental impacts, and livelihoods, as well as their trade-offs. The calculation of the global economic value of farmed animals is also a critical component for the Global Burden of Animal Diseases (GBADs) Programme (Rushton et al., 2021) where it is needed as the basis for a global animal disease burden estimation.

The aim of this study was to approximate the global economic value of farmed animals using available data. The Total Economic Value (TEV) concept (Bateman et al., 2002; National Research Council, 1999; Pearce et al., 2006) was adapted to the context of this study as a baseline framework to illustrate the types and scale of economic value that farmed animals generate (see section 2).

Due to the lack global data for a range of economic value types, e.g., finance/insurance service value, non-market values, the scope of the analysis was limited to the direct use value, that is the market value of live farm animals and primary outputs that they produce, e.g., meat, eggs, milk. The analysis focussed on terrestrial livestock species including cattle, pigs, sheep, chickens, goats, horses, mules, buffalos, and other domesticated species, and farmed aquatic animal species (e.g., carp, prawns, tilapia, salmon, oysters, mussels, and other species). While a comparison with the market value of crops was undertaken in this study to identify the relative economic importance of the farmed animal sector, a detailed assessment of the TEV for crops was beyond the scope of this study.

## 2 An economic value framework for farmed animals

Economic value is a concept that describes the weighting or importance that individuals place on something (e.g., goods, services, experiences), reflecting the benefit or utility (e.g., pleasure, gratification, satisfaction, virtue) that they gain from it (Viner, 1925). Importantly, the terms ‘value’ and ‘price’ are not synonyms (Viner, 1925). Price is a measure of the monetary amount at which something is exchanged in a market (i.e., market value) (Gowdy, 1997). Yet, the prevailing market price may not reflect the complete economic value of something to individuals; in some instances, markets and subsequently market values may not exist (Gowdy, 1997; Martin-Collado et al., 2014).

**Fig. 1:**
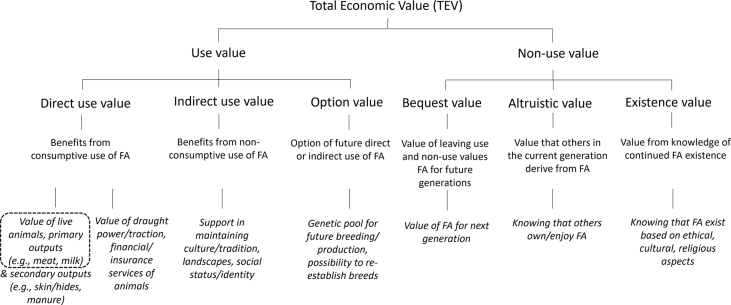
Total Economic Value of farmed animals.

Source: Adapted from Bateman et al. (2002), Pearce et al. (2006), Nyariki and Amwata (2019), Hansson et al. (2018). Notes: FA for farmed animals. Text in italic font are examples. The dashed box indicates the economic (market) value component that is being approximated in this study at global scale, based on available data (details provided in section 3).

The TEV concept (Bateman et al., 2002; National Research Council, 1999; Pearce et al., 2006) has been used widely for cost-benefit analyses to assess the economic value that society derives from environmental assets (e.g., Bilmes and Loomis, 2019; Deloitte Access Economics, 2017; Emerton, 2018). In this study, we adapted the TEV concept to the context of farmed animals to demonstrate various categories of the economic value which farmed animals provide to individuals and society (Fig. 1).

The TEV comprises the sum of farmed animal’s *use value* and *non-use value* (adapted from Bateman et al., 2002; Pearce et al., 2006, Fig. 1). The use value of farmed animals can be defined as their benefit to individuals (or society) through their direct interactions with live farmed animals, animal outputs and services that farmed animals provide (e.g., sale/purchase of livestock and outputs, use of animals for cultural ceremonies) (adapted from Bateman et al., 2002). The use value of farmed animals can be further sub-categorised into direct use value, indirect use value and option value (e.g., Nyariki and Amwata, 2019; Zander et al., 2013).

*Direct use* values can be defined as the economic value derived from the consumptive use of goods. This includes the value of a) live animals as a product that is exchanged in a market at a price which is derived from demand and supply, b) primary outputs, e.g., meat, milk, eggs, and secondary outputs, e.g., wool, horn, skin/hides and manure, which are exchanged in a market at a price, and c) services provided by animals such as draught power/traction, insurance and financial services, e.g., creation and storage of wealth, risk management function, for which markets exist (e.g., Ayalew et al., 2003; Martin-Collado et al., 2014). Insurance/finance services are specifically important for livestock owners in lower income countries where animals also offer security against contingencies, e.g., a substitute for insurance premiums; and are means of financing, e.g., animals as assets that can be liquidised into cash (Moll et al., 2007). To estimate the insurance/finance value, comparable insurance/finance options can be considered as a reference (Moll et al., 2007). Hence, the direct use value of farmed animals arises from concerns about productivity and profitability of animal production that has a monetary exchange value (e.g., Hansson et al., 2018; Lagerkvist et al., 2011).

*Indirect use* values are benefits derived from non-consumptive use of goods and services that farmed animals provide. These include for example animals' support in maintaining cultural, traditional, and religious values (e.g., identity) and landscapes (e.g., extensive grazing to reduce fire risks, control of invasive grass species) (Davies, Wollstein, Dragt and O'Connor, 2022; Martin-Collado et al., 2014; Szűcs et al., 2012).

Another use value of farmed animals is the *option value* defined as the value or benefit derived by society from the future use of farmed animals, e.g., genetic breeding pool for future production (Drucker et al., 2001; Ejlertsen et al., 2012; Nyariki and Amwata, 2019; Zander et al., 2013).

The non-use value of animals refers to their intrinsic or passive value to society (adapted from Bateman et al., 2002). This includes: a) *bequest value* – the economic use and non-use value that future generations may derive from farmed animals; b) *altruistic value* – the value that others in the current generation derive from animals; and c) *existence value* – the value from the knowledge of the continued existence of farmed animals, e.g., display of status (adapted from Bateman et al., 2002; Pearce et al., 2006). While a range of studies cite the existence of non-use values of farmed animals, specifically for livestock in traditional and pastoral systems (e.g., Gandini and Villa, 2003; Marsoner et al., 2018; Moll, 2005; Pearce et al., 2006), empirical studies that attempted to quantify these values are limited, e.g., Hansson and Lagerkvist (2015) and Bennison et al. (1997).

More detail about these value categories is provided in the supplementary material.

## 3 Data and methods

### 3.1 Data

The TEV framework (Fig. 1) was used as the baseline to identify existing data for the approximation of the global economic value of farmed animals between 1998 and 2018. The global context of this study (i.e., 181 countries) and the associated scale of data requirements implied that global indirect use value, option values and non-use values of farmed animals could not be included in the analysis. Such data has not been collected and would require a dedicated research programme to address the data need.

Global data for direct use values of terrestrial livestock and aquatic farmed animals such as the production quantities and prices or market value (i.e., the product of quantity and price) of live animal stocks and animal outputs (i.e., meat, fish, milk, eggs) per country were available from the Food and Agriculture Organization (FAO) of the United Nations' statistical livestock database (hereafter FAOSTAT) and aquaculture database (FAO, 2021a, 2021b). These datasets offer the most comprehensive compendium, including timeseries, of global terrestrial livestock and aquatic farmed animal statistics, disaggregated by species and country, which can be used to approximate their direct use or market value (Fig. 1, dashed box).

Key variables available from FAOSTAT for terrestrial livestock species and their production outputs used for the study analysis included: annual population size of producing animals (recorded in FAOSTAT as “Producing Animals/Slaughtered”, which is a single estimate of the total number of animal heads that generate certain animal products), average annual farm-gate price, and average annual market value of outputs (i.e., already calculated market value based on price and quantity by output type and country) (FAO, 2021a).

The list of livestock species included in the analysis of live animals included: asses, birds (non-poultry, including birds in shell), buffalos, camels, other camelids, cattle, chickens, ducks, game, goats, geese, horses, mules, rodents, pigs, sheep, and turkeys (FAO, 2021a).

Biomass conversion factors for terrestrial livestock (FAO, 2021c) were used to transform the number of animals into a weight equivalent; this was necessary to match live animal price data, which is reported in tonnes/year. FAO liveweight and dressing percentage (FAO, 2021c) were applied in cases when primary conversion factor data were unavailable. For years in which the FAO reported carcass yield or tonnes of output and number of animals slaughtered, only FAO’s dressing percentage was used alongside carcass yield to calculate average live body weight.

Data for primary outputs generated from these animals included meat, milk and eggs (FAO, 2021a). While quantities of produced secondary outputs such as offal, fat, hides/skins were available for livestock species in FAO (2021a), price data for these outputs were missing. Therefore, secondary outputs produced from terrestrial livestock could not be included in the analysis.

Data for aquatic farmed animal species were taken from FAO’s Fisheries and Aquaculture Division and included only output quantities (i.e., presented as product forms such as whole fish, fillet, in shell) and the market value of outputs (i.e., previously calculated market value based on price and quantity by species and country) (FAO, 2021b). The FAO data set comprised 630 aquatic farmed species, including key species such as carp, prawns, tilapia, salmon, oysters and mussels (FAO, 2021b).^2^ Aquatic farmed species such as pearls, mother-or-pearl and shells were excluded from analysis since they are not used as food. The value of live aquatic farmed animals could not be included in the analysis, given the absence of information about stock quantity, prices and other metrics that could be used to derive estimates on a global scale.

Global data on the value of growing crops, which is typically valued at the cost of inputs only (e.g., Brashears, 2021), was not available with global coverage, nor was equivalent data for livestock. Global crop output data, e.g., harvested/marketed crops from FAOSTAT (FAO, 2021a) were used in the analysis to provide information about the relative importance of the economic value generated by farmed animals in the context of global food production for human consumption.

The economic values approximated in this study represent the annual market value of farmed animals and their primary outputs in constant (inflation adjusted) 2014–2016 US Dollars (USD). This implies that any costs (e.g., feed, labour, fuel, veterinary services, medicine) that were incurred during the production of farmed animals by the producer are reflected in the farm-gate price at which animals are sold. The same applies to the market value of animal and crop outputs (e.g., meat, eggs, milk, grains), costs that were incurred by value adding processes (e.g., animal slaughter, harvest, processing) are assumed to be reflected in the available market value data (FAO, 2021a, 2021b). Social costs of farmed animal and crop production (e.g., greenhouse gas emissions, land degradation, loss of biodiversity) are not mirrored in these market values. Potential seasonal price variations of live animals and outputs (i.e., animal, crops) are assumed to be accounted for by the available annual average price data. For analysis in which the economic value in a specific period, e.g., 2018, was compared across countries, prices needed to be adjusted to account for purchasing power parity (PPP). Conversion rates available from The World Bank (2022) were used for the adjustment of these values to the International Dollar unit. To estimate the relative economic importance of farm animal production by country, human population data from The World Bank (2021) was used.

More detailed information about data used in the analysis is provided in the supplementary material.

### 3.2 Methods

Given available data, the focus of the analysis was on approximating the global direct use or market value of live terrestrial animals, hereafter referred to as asset value, and the primary output market value generated by terrestrial animals and farmed aquatic species (see Fig. 1, dashed box).

In this study, live farm animals were defined as assets, that is a resource controlled by an entity, e.g., smallholder, business, country; as a result of past events, e.g., production inputs and management, from which future economic benefits are expected to flow to the entity (Petzke, 1998). The market price, e.g., farm gate price, of live animals was used as a basis for asset valuation (Petzke, 1998).

The output value was considered as the quantity of primary outputs/offtake from farm animal production which includes meat from terrestrial and aquatic animals, eggs, and milk; multiplied by its market price.

Since the study aimed to compare the market value of farmed animals and their outputs with the market value of crops, these definitions also applied to crops with a minor variation for asset values. For example, the crop asset value was considered as growing crops, e.g., crop on field, valued at their costs of production; and unsold crops in inventories, e.g., grain in pools/bins, valued at its market price (Brashears, 2021). The crop output value comprised harvested crops that were sold in the market at the market price.

#### 3.2.1 Calculation of the asset value

The asset value was estimated using the following equation:

(1.1) $$\sum_{i,j}x_{i,j}p_{i,j}$$

where $x_{i,j}$ is the live terrestrial animal population (i.e., number of heads) for livestock species *i ϵ* (asses, birds, buffalos, camels, other camelids, cattle, chicken, ducks, game, goat, geese, horses, mules, rodents, pigs, sheep, turkeys) for country *j ϵ J* including 181 countries (see FAO (2021a)). *p*_*i,j*_ is the associated average live weight unit price (farm gate price) for the respective livestock species in a specific year.

A conversion from live animal numbers (i.e., head) into a weight unit equivalent (i.e., tonnes) using conversion factors, $f_{i,j}\text{,}$ for livestock species in each country was needed to match price data which was only reported as price per tonne at farm gate level in FAO (2021a). The liveweight unit equivalent or biomass are expressed as:

(1.2) $$\sum_{i,j}x_{i,j}f_{ij}$$

The live weight conversion factors, $f_{i,j}\text{,}$ for each terrestrial animal species were derived from the FAOSTAT dataset using the carcass yield for each terrestrial animal species (Enahoro et al., 2018).

The derived biomass of live animals reported in the data for a prevailing year was then multiplied by the average farm gate price for each livestock species, *p*_*i,j*_.

(1.3) $$\sum_{i,j}x_{i,j}f_{ij}p_{i,j}$$

The global asset value (i.e., total value of live animals) of each livestock species and subsequently all species and for each year was calculated by summing the value of all countries.

Due to the lack of global data for aquatic assets and crop assets, an estimation of the value of live aquaculture fish stocks and crops in pools and on field could not be included in the analysis.

#### 3.2.2 Calculation of the output value

The calculation of the market value of primary outputs or offtake (e.g., meat, milk, eggs) from farmed animal production (see Fig. 1, dashed box) in a year is represented by:

(2) $$\sum_{k,j}y_{k,j}v_{k,j}$$

with *y*_*k,j*_ as the output quantity for commodities *k ϵ (meat, milk, eggs)*, where meat includes terrestrial and aquatic animal sources, and associated output market prices *v*_*k,j*_ (based on FAO, 2021d). The total global value for each output type and for each year was calculated by summing individual output market values for all countries.

Equation (2) was also used to estimate the output value of global crop production (e.g., maize, rice, wheat, fruit, and vegetables; see full list in the supplementary material) for comparison with values generated by the global farmed animal sector.

#### 3.2.3 Calculation of the combined asset value and output value

To derive the combined market value of farmed animal assets and output values for each species in a specific year, the sum of equation (1.3) and equation (2) for all countries was calculated using:

(3) $$\sum_{i,j}^{J}x_{i,j}f_{i,j}p_{i,j}+\sum_{k,j}^{J}y_{k,j}v_{k,j}\text{.}$$

This equation broadly aligns with the method presented by Jarvis (1974), Li et al. (2018) and Hennessy and Marsh (2021) for estimating value using the market value of live animals and outputs that they generate. Yet, this approach includes the risk of double counting the present (potential) future output value as part of a farmed animal’s current asset market value. This is the market value of a live animal at a specific time (e.g., a steer at 12 months), which also includes a value describing some proportion of the animal’s future output (e.g., meat). Furthermore, there may also be an overlap in the number of live animals and animals that may have been slaughtered for meat (output) within a year. Given these uncertainties, we assume that the true market value of farmed animals lies between the sum of global market asset value of farmed animals (equation (1.3)) and the overall total of the global market asset value plus the global output value (equation (3)). More detailed explanations for all equations are provided in the supplementary material.

## 4 Results

### 4.1 Annual asset value and output value of farmed animals

**Fig. 2:**
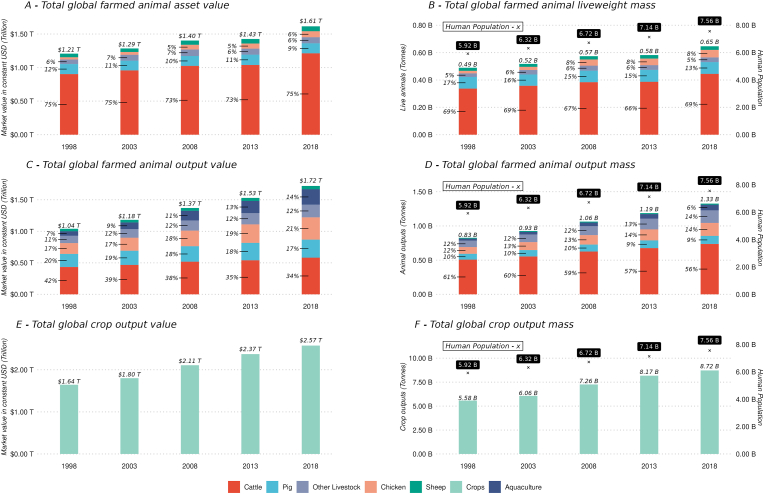
Global asset value, output value and mass of farmed animals and crops between 1998 and 2018 Notes: Aquaculture estimates are not included in asset value as global data are unavailable. ‘B’ for billion, ‘T’ for trillion. Values are presented in constant 2014–2016 USD. Sources: FAO (2021a), FAO (2021b).

The cattle sector has historically contributed the largest proportion to the global asset value (and liveweight mass) of farmed animals (i.e., more than 70% between 1998 and 2018), followed by the pig (around 10%) and chicken (around 7%) sectors (Fig. 2, pane A). The contribution of sheep and other animal species (e.g., goats, camels, horses) to total global livestock asset value has remained relatively small over time (around 5%, respectively).

The global total asset value, which includes all livestock assets, increased around 24% from 1.21 to 1.61 trillion USD (i.e., constant 2014–2016 USD) between 1998 and 2018 (Fig. 2, pane A). This trend is reflected by the global rise in the total liveweight mass of farmed animals (24%) as well as by the rise in the human population (22%) and subsequent demand for food that farmed animals generate (Fig. 2, pane B).

The global total output value of farmed animals is also dominated by the value that cattle outputs generate (34% in 2018), followed by chickens (21%), pigs (17%), aquaculture (14%), other livestock (12%), and sheep (2%). The proportion that each animal species contributes to total output value and total output mass has not changed much over time, except for aquaculture which increased from 7% to 14% between 1998 and 2018. It should also be noted that animal output mass (e.g., weight of meat, fish, eggs, milk) exceeded animal liveweight mass for all time-periods (Fig. 2, pane B and D), due to the inclusion of short production cycles species (e.g., pigs, chicken) and the weight of milk.

The total global output value of farmed animals, including meat (livestock and fish from aquaculture production), milk and eggs, has increased around 40% from 1.04 to 1.72 trillion (constant 2014–2016) USD during 1998–2018 (Fig. 2, pane C). The increase in output value between 1998 and 2018 has been greater than the increase in asset value, with livestock outputs having greater value than livestock assets in 2013 and 2018. The relatively faster increase in the total global output value can be explained by the output mass which has increased by about 38% (i.e., from 827 to 1326 million tonnes, including aquatic farmed species), while the liveweight mass only increased by 24% (i.e., from 490 to 646 million tonnes) during this period. The relatively high growth rate in output mass may be explained by the adoption of improved farm management practices (e.g., housing, animal health, feed composition that result in higher meat, egg, milk yields) and output generating technologies (e.g., carcass processing equipment and subsequent carcass yields).

The relative economic importance of the farmed animal sectors can be assessed by comparing its value to the global total crop output value and crop output mass over time (Fig. 2, pane E and pane F). Global crop output value and crop output mass have historically exceeded output values and mass for farmed animals. However, considering a value per mass unit, which is the ratio of market value and output mass (tonnes), live animals (e.g., 1.61 trillion USD/0.46 trillion tonnes equal 2476 USD/tonne in 2018) and animal output (1293 USD/tonne in 2018) appear to exceed the value of a unit of crops (295 USD/tonne in 2018). These estimates suggest that a tonne of animal outputs has a higher market value than a tonne of crop outputs. Yet, these aggregated results may not hold true at similar scale for comparisons of specific animal output types (e.g., meat, milk, eggs) and high value crop outputs (e.g., fruit, nuts).

### 4.2 Combined global asset and total output value

**Fig. 3:**
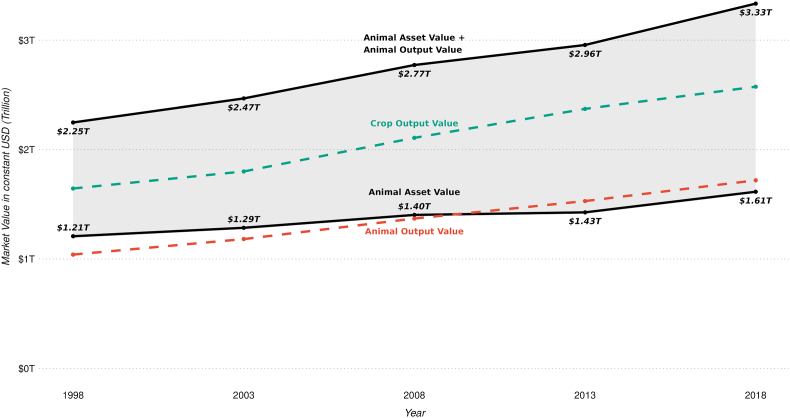
Approximation of the true global market value of farmed animals and global crop value (1998–2018) Note: The shaded area represents the value range within which the true total global economic (market) value of farmed animal may be found. ‘T’ for trillion. Values are presented in constant 2014–2016 USD. Sources: FAO (2021a), FAO (2021b).

Adding up the annual global asset value and annual output market value provides an approximation of the annual total global economic value of farmed animals (equation (3), Fig. 3). For example, for 2018 the total global economic value of farmed animals was estimated at 3.33 trillion USD. As outlined in the methods section, the estimate for the total global economic value of farmed animals likely includes some degree of double counting of the current potential future output value in a prevailing year as part of a live animal’s current asset market value. Therefore, the ‘true’ total global economic value of farmed animals is expected to be found within the range between the global total asset value as a lower bound and the sum of global total asset value and total output value as an upper bound (shaded area in Fig. 3). Fig. 3 also shows that the global total crop output value falls within the range of the true total global economic value of farmed animals.

### 4.3 Spatial distribution of the asset and total output value

**Fig. 4:**
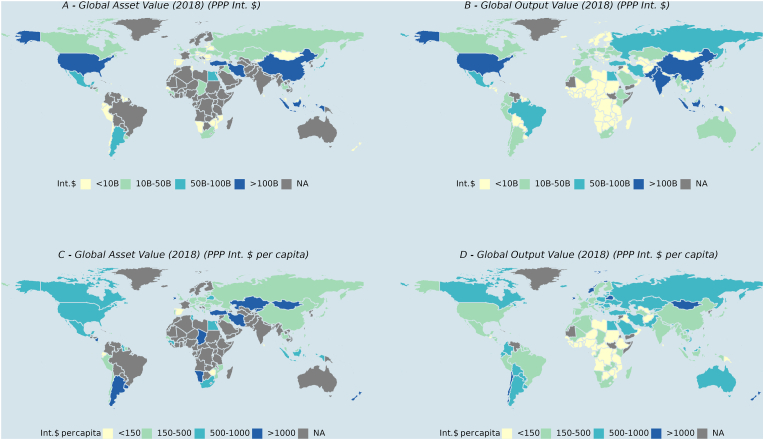
Spatial distribution of combined global farmed animal asset value and output value in 2018 Notes: Values are presented in International Dollars (Int. $) to account for purchasing power parity (PPP). NA indicates that data were not available including for either stocks/quantities, price/value or local currency unit/standard local currency data for 2018. ‘B’ for billion USD. Sources: FAO (2021a), FAO (2021b), The World Bank (2022).

The USA, Turkey, Iran, China and Indonesia generate the highest value for live animals globally (Fig. 4, pane A). The USA, China and Indonesia also lead the generation of output values from farmed animals, together with Pakistan and India (Fig. 4, pane B). A relatively low value of assets in absolute terms is generated in countries such as Chile, Peru, Namibia, Spain, and Mongolia. The map also shows many data gaps for 2018 (‘NA’), including for countries with large herd populations. These include actual missing observations for many countries (e.g., number of animal head for France, prices for India). In some cases (e.g., Brazil, Australia), prices for comparative analyses are available in constant USD but not as local currency units/standard local currency units; these are necessary to convert prices into International Dollars (PPP adjusted values).

As an indicator to compare the economic importance of the farmed animal sector across countries, the value of animal assets and outputs per country was divided by each country’s human population. The results suggest that Argentina, New Zealand, Ireland, Namibia, Chad, Turkey, Iran, Kazakhstan and Mongolia generate the largest global market values per human capita for live animals (Fig. 4, pane C). Mongolia, Ireland, New Zealand, Belarus and Norway are also amongst countries with the highest global output value per human capita, alongside Albania and Armenia (Fig. 4, Pane D), indicating the high economic importance of animal outputs in these countries. The lowest output value in absolute and per capita terms is generated in low-income countries in Africa, Iraq, Uzbekistan, Afghanistan, and Papua New Guinea. The supplementary material offers additional results describing the spatial distribution of asset values by livestock species.

### 4.4 Change in market value by country over time

**Fig. 5:**
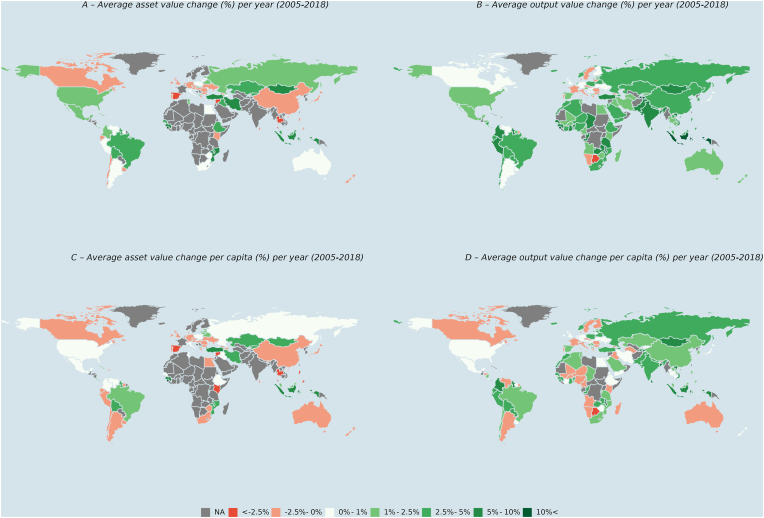
Average annual changes in asset value and output value between 2005 and 2018 Notes: NA indicates that data were not available including for either stocks/quantities, price/value or local currency unit/standard local currency data. All values in constant 2014–2016 USD. Sources: FAO (2021a), FAO (2021b), The World Bank (2021).

While Fig. 2 illustrates changes in the total global market value of farm animal assets and outputs, Fig. 5 describes the average annual livestock asset value change (%) between 2005 and 2018 per country (Fig. 5, pane A). The 2005–2018 timeframe was selected for this analysis as the data quality (e.g., more complete observations per county) for this period was higher compared to production years preceding 2005. These results suggest an average annual increase in livestock asset values of 5% or more in Indonesia, Mongolia, Tajikistan, Turkey, Ethiopia, Mozambique, Sierra Leone, Guinea, Guinea-Bissau and Brazil. These average changes in the annual livestock asset values within this 13-year period can be attributed to changes in animal populations and market prices dynamics.

A decrease in average annual livestock asset value can be observed for Thailand, Syria, Canada, Ecuador, Chile, Kenya, New Zealand, Japan, China, some EU countries, and the United Kingdom. This suggests that changes in livestock asset values over time may not be predominantly driven by the economic development of a country, and that other aspects also affect these trends (e.g., structural economic change, conflict). Fig. 5, panel C illustrates the change in asset values relative to the human population in each country. For countries such as the USA and Russia this implies that the value generated is in line with the increase in the human population, potentially contributing to food, nutrition, and asset wealth creation. For other countries such as Brazil, Turkey, Iran, Kazakhstan, Mongolia and Indonesia, the results imply that the asset value of livestock likely provide wealth beyond the populations’ needs, e.g., due to exports. Data gaps for many African and Arabic countries as well as for India prevented their inclusion in this analysis.

The map shown in Fig. 5, Pane B describes the average annual change in livestock and aquaculture output values between 2005 and 2018. The results suggest a positive average annual change in the output value for many low-income (e.g., Mozambique, Uganda, Nigeria, Afghanistan), low middle-income (e.g., Indonesia, Mongolia, India) and upper middle-income countries (e.g., Peru, Brazil, Chile). For high income countries, the annual change in the output values was smaller (e.g., USA, Canada, UK, Germany, Finland, Spain, Australia).

These results may be explained by the higher growth rate in the output volume in lower income countries compared to high income countries during this period (see supplementary material). Potential drivers for the higher growth in output volume in lower income countries may be a higher demand due to higher human population growth rates compared to high income countries (FAO, 1997; The World Bank, 2023). Furthermore, increased food affordability due to rising incomes, specifically in lower middle and upper middle countries, may have contributed to the increasing output demand in lower income countries (Enahoro et al., 2018).

On the other hand, changing diets in high income countries, e.g., less meat, more fats, sugar, whole grains, fruit and vegetables may have contributed a lower growth in the demand for animal outputs and subsequently the output value (Mathijs, 2015).

Yet, Fig. 5 (Pane B) also illustrates that there are variations within country income groups, for example, for some low-income countries such as Namibia, Botswana, South Sudan, Libya, and Syria a decrease in the output value over time was recorded. These results could be explained by country specific characteristic (e.g., conflict) and data availability/quality for the period under review.

Normalised by the size of the human population, the average annual change in output values shows that most low income (e.g., African) countries experienced a decrease in output value per capita over time (Fig. 5, pane D). This result suggests that the output value relative to human population has declined for these countries over time. However, this appears to also the case for higher income countries such Argentina, Canada, Sweden, and several EU countries.

## 5 Discussion and conclusion

The results of this study offer previously unidentified insights into global patterns and trends in the economic (market) value of farmed animals for global human society. We show that the combined asset and output value of livestock is comparable to the output value of crops and highlight regional and species diversity in how livestock systems are valued. Our analysis also emphasizes various data gaps, which limit the scope of these analyses at the global scale.

The findings of this study provide an important benchmark for the GBADs programme to better understand the benefits that farmed animals offer society, e.g., asset and output value within the TEV concept; compared to the economic burden of animal diseases (e.g., Gilbert et al., 2023; Rasmussen et al., 2022). The study outcomes offer a baseline for discussions among peak bodies of the livestock and aquaculture sectors, national governments and intergovernmental organisations, e.g., World Organisation of Animal Health, FAO; about potential investment needs in improved animal health, the link between animal health and the future of the global food system as well as the benefits that animals provide society beyond their direct market use value.

A key outcome of this study was the quantification of both the asset and output value of livestock. These values also capture some of the direct service use and indirect use values of livestock (see Fig. 1). For example, while pork consumption in Timor Leste is very low, the market value of live animals is very high, reflecting their high socio-cultural value for ceremonies (D. Smith, Cooper, Pereira, & da Costa Jong, 2019). Furthermore, the insurance and finance service value of live animals and their outputs in lower income countries can account for up to 6–10% of the herd value, respectively (Moll et al., 2007). The social status of farmed animal owners in lower income countries can reflect up to 3% of animals’ asset and output value (Moll et al., 2007).

Findings from this analysis also suggest that the global asset value of farmed animals and the output value have been relatively similar in their absolute terms during 1998–2018 but differ between species. Larger and long-lived species such as cattle and sheep have relatively higher asset values, whereas the market value of small and short-lived species (e.g., chickens, pigs) is primarily captured in their outputs.

The results showed that the true global economic value of farmed animals lies between the output value and the sum of the asset value and output value (Fig. 3, shaded area). The lack of global data describing national herd structures (e.g., age, breed, gender) and production parameters (e.g., salvage value, depreciation rate, investment rate, input costs) currently prevents further narrowing of the range within which the ‘true’ global market value of farm animals can be found. However, based on the present analysis we expect that the total global market value of farmed animals would be similar to the global output value of crops. The lack of data about the global crop asset values, e.g., crop production costs, prevents a comparison of the total market value of famed animals and crops. While the TEV concept has not yet been adapted to crops, it is likely that value categories of crops and their actual values as well as their weightings differ to the ones for farmed animals (Fig. 1), for example, due to differing resource and tending/labour requirements, equipment, and investment among other. However, such comparison remains subject to future research.

There are considerable spatial differences in the market value of farmed animals (Fig. 4) which is reflected in the intensity of global animal production (e.g., Gilbert et al., 2018). Countries with high asset and output values typically have a comparative advantage for farm animal production (e.g., availability of land, water, labour, technology/skills) (e.g., Cai and Leung, 2008; Cattaneo, 2008). These countries commonly engage in the international trade of live animals and animal outputs (e.g., USA, Brazil, Canada, Australia) (FAO, 2021a, see trade table) particularly if they have the wealth, governance and political influence needed to obtain widespread export market access. Trade in both live animals and animal outputs positively impacted food and nutrition security in importing countries but can also result in the spread of animal diseases (e.g., Rautureau et al., 2011). While we have not captured trade flows in our analysis, these are important and should be factored in (e.g., as value added post farm-gate) when determining the global market value of farmed animals (Erokhin et al., 2021).

The results also demonstrate that changes in the economic value of farmed animals over time vary depending on the country (Fig. 5). Such changes in the economic value of animals and their outputs can be affected by changes to animal stock management/governance (e.g., herd/stock structure change, on-farm investments, disease prevention and treatment management), environmental conditions (e.g., drought, soil erosion), market dynamics (e.g., food market trends in general which can affect the market value of live animals and output), and political stability (e.g., conflict affecting stock size, inflation) (Hendrix and Haggard, 2015; Leister et al., 2015; Maystadt and Ecker, 2014).

A further finding from this study is that the cattle sector holds a dominant role in global farmed animal production in terms of its market value, liveweight mass and output mass compared to other farm animal species (Fig. 2). However, the aquaculture sector has grown significantly in the past decade reflected in its increasing market value. This result is supported by Naylor et al. (2021) who recently undertook an analysis of growth dynamics within the global aquaculture sector. This finding can be attributed by an increasing global demand for fish products for human and animal consumption which is linked to the growth of global human population (FAO, 2022). The increased supply of aquaculture products is also linked to the adoption of improved technologies, e.g., breeding, feed input, enhanced farm management and value chains over the past decade (Naylor et al., 2021). These dynamics are reflected in the gradual growth of the output value that the aquaculture sector generated.

Given the globally important economic role of farmed animal production (Fig. 3), it is imperative that animal health can be maintained to allow the continuous supply of these contributions to society. Consequently, targeted global and national investments to address the global burden of animal diseases (e.g., prevention, treatment, biosecurity) are needed, specifically in low-income and lower-middle income countries where improved animal health (e.g., longer life, higher productivity) is closely linked to sustaining livelihoods (e.g., Homewood et al., 2006). Improved global animal health will likely also contribute to an increase in animal productivity and a reduction in the environmental footprint (Bellet et al., 2021).

Furthermore, the market value that is generated from farmed animal production and its global spatial distribution (Fig. 4) also needs to be considered in conjunction with social costs of animal production. Countries that generate a very high economic value (e.g., Brazil, China, India and USA) should be key contributors to mitigating not just local and national social costs (e.g., soil erosion, displacement of biodiversity), but also global social costs of farmed animal production (e.g., deforestation, greenhouse gas emissions specifically in the case of intensive cattle feedlot systems) (e.g., Herrero et al., 2013; Tabassum et al., 2016).

Due to the lack of global data about potential indirect use, option and non-use values that farmed animals may generate, it is currently impossible to judge whether their total net economic value is higher, lower or about the same compared to market value estimates in this study. The limited research and methods available, specifically in the area of indirect use and non-use values of farmed animals, suggests opportunities for further explorations. An improved understanding about the indirect use and non-use values of farmed animals is important to fully capture these types of economic value that farmed animals provide global society, and specifically in countries where animals are part of livelihoods.

There are a range of limitations to this study’s analysis, including issues around data availability (e.g., price data, PPP conversion rates) and quality for an economic analysis of asset and output market values at global scale. For example, within the FAOSTAT livestock dataset for the 2018 production year alone, there are 3147 missing observations globally, demonstrating the scale of data gaps. The lack of data for several countries, including missing records of stock (e.g., head), output volume and price/value or both for specific production years and animal species, contributes to a possible underestimation of the market value for assets and outputs. The lack of data about livestock breeds, age structure, salvage value, body condition and depreciation rates of herds at national and sub-national scale limit more detailed economic value assessments. Furthermore, the different life cycles of animal species were neglected in the estimation of asset values. This is because the FAOSTAT data (FAO, 2021a) only offers the total number of heads per species present during the course of a year and does not disaggregate these into live and slaughtered animals. While neglecting the life cycle dynamics in the estimation could bias the results when focussing on a specific production year (e.g., over- or underestimation of asset values), we assume that this potential bias reduces when several production years are considered in the same manner (e.g., same potential bias applies for each individual year in the time series, meaning that the calculation of asset value for each individual year includes the same potential bias).

Improving the availability of comprehensive and reliable data is an essential step for addressing the challenges that the global food sector is facing, such as how to manage trade-offs between increasing demand and environmental impact, and how to invest in prevention or better management of animal diseases. Therefore, investment and global collaboration in data collection and ‘FAIRS’ness of data (Findability, Accessibility, Interoperability, Reusability, and Secure) is critical to provide improved data driven decision making within the farm animal production sector (Stacey et al., 2022; Wilkinson et al., 2016).

## Declaration of competing interest

The authors declare the following financial interests/personal relationships which may be considered as potential competing interests: Corresponding author and all co-authors report that financial support was provided by Bill and Melinda Gates Foundation. Corresponding author and all co-authors reports financial support was provided by Commonwealth and Development Office of UK government.

## Data Availability

The data source has been shared as an external link. FAOSTAT (various, specified in manuscript) (Original data)

## Footnotes

2 FAO (2021b) provides the full list of aquatic farmed animals included in the analysis, see ‘CL_FI_SPECIES_GROUPS’ under category Yearbook Group “Fish, crustaceans and molluscs, etc.".
