# Supplementary material for: Approximating the global economic (market) value of farmed animals
Source: Glob Food Sec. 2023 Dec;39:100722. doi: 10.1016/j.gfs.2023.100722 (PMC10714036; doi:10.1016/j.gfs.2023.100722)
Supplement: Multimedia component 1 [file mmc1.docx]

**Supplementary Material**

This document provides additional information about the conceptual framework which is used as a baseline (section 2), data (section 3.1), and methods (section 3.2) used to approximate the global economic value of farmed animals, along with supplementary results (section 4).

**Section 2: An economic value framework for farmed animals**

The Total Economic Value (TEV) concept (Bateman et al., 2002; National Research Council, 1999; Pearce, Atkinson, & Mourato, 2006) has been used widely for cost-benefit analyses to assess the economic value that society derives from environmental assets such as reefs, wetlands, national parks and from endangered species as a basis for decision making about the management of these assets (e.g., Bilmes & Loomis, 2019; Deloitte Access Economics, 2017; Emerton, 2018).

The option value of farmed animals includes the value of the genetic animal breeding pool for future production, which is specifically important for rare or threatened livestock species and for improved breed resilience such as for disease resistance, reduced enteric methane, adaptation to heat stress, and for improvements in efficiency of resource use (e.g., Drucker, Gomez, & Anderson, 2001; Zander, Signorello, De Salvo, Gandini, & Drucker, 2013). Preservation of indigenous culture/heritable characteristics of breeds (e.g., cultural distinctiveness of breeds such heat stress resistance, ability to withstand droughts, traditional knowledge of handing these breeds) is another example for the option value of farmed animals (Ejlertsen, Poole, & Marshall, 2012; Zander & Drucker, 2008). Studies that have estimated the option value of farmed animals include Nyariki and Amwata (2019) and Zander et al. (2013).

Important for the estimation of the TEV in the farmed animal context is the consideration of the net economic value (i.e., net benefit derived from private/public benefits less private/public costs) which requires the inclusion of perspectives from various stakeholder groups (e.g., producers, consumers, interest groups).

There is no single methodology to estimate the TEV (Figure 1, main article). Estimation of direct use values (i.e., market value of live animals or meat) and some indirect use values (e.g., landscape maintenance services) of farmed animals is typically straightforward using existing market data and relatively simple methods (e.g., product of market price and production quantity, opportunity costs). However, for the estimation of other value categories, data (e.g., option value, non-use values) is typically not readily available and may require complex primary data collection and analysis methods. These methods can include choice experiments (e.g., willingness to pay to ensure breed continuation), travel cost analysis (e.g., expenses that individuals are willing to pay to experience farmed animals), contingent valuation and other approaches (e.g., Conte, 2013; Hensher, Rose, & Greene, 2015; Whitehead & Haab, 2013). The choice of data collection and analysis methods also depends on the specific research question and context.

**Section 3.1: Data**

While the Gridded Livestock of the World (Robinson et al., 2011) offers an alternative dataset that disaggregates terrestrial livestock populations by production system, this data does not include information on livestock products, is only available for two time points (2010, 2015), and excludes species such as camelids, which are regionally important. There is also no equivalent disaggregation for aquatic species. Therefore, the Gridded Livestock of the World dataset was considered as insufficient to address the aim of the present study.

The farm gate price (i.e., first-point-of sale at which the producer participates is the market as a seller for their farmed animals) is the amount receivable by the producer from the purchaser for a unit of a goods minus any value added tax, excluding transport charges (FAO, 2021a).

Data from each country’s national records (e.g., statistical bureau) for animal populations, herd age and breed structure, salvage value, depreciation rate (i.e., loss of live animal asset value over its useful life), animal prices and generated output statistics were also considered. However, such data are not publicly available for most countries and if so, collection methods and variables can differ across countries (e.g., aggregation of livestock species) which prevents a comparison of these variables across countries.

No data for live populations or prices of live aquatic animals were available either from FAO or from countries’ national records. We considered the use of output data from aquatic animal production, converting these into live weight equivalents for aquatic animals. Such data would provide a crude estimate of the total number of slaughtered animals per species (e.g., neglecting losses, stock age structure). However, indicative factors for converting product weight to live weight are globally only available for a limited number of fish species and only for wild-caught fish (FAO, 2021c). Furthermore, prices of live fish in aquaculture systems could be significantly different to output prices (e.g., due to value adding such as gutting, filleting) and may not follow similar relationships between asset value and output value as for terrestrial livestock species (i.e., they are different products, different production costs per unit may apply). The asset value of aquatic farmed animals could not be included in the analysis, given the absence of information about stock quantity, prices and other metrics that could be used to derive estimates on a global scale.

The focus of the analysis was on primary crops and included food crops only (i.e., non-food crops such as rubber were excluded). Processed crops were excluded as these are specialised products such as beer from barley, molasses, oils (e.g., coconut, palm, sunflower) and wine which are considered here as not vital to ensure food security and nutrition. The Appendix A.1 provides a list of countries, livestock types, aquaculture species, outputs and crops included in the analysis.

**Section 3.2 Methods**

***Equation 1.1 (asset value)***

The average farm gate price is assumed to reflect price components such as animal characteristics (e.g., sex, age, breed, condition, size), cost of production inputs, seasonal supply and demand variations, cross-price dynamics (e.g., elasticities between cattle and goat quantities and prices), transportation costs to markets, market location, a profit margin (e.g., Barrett, Chabari, Bailey, Little, & Coppock, 2003).

Notably, equation 1.1 does not reflect the value generated through actual market sales of farmed animals in a country in specific year (current value), but their asset value which can include actual sale value of animals that were sold and the value of animals that have not been sold in a market. Since no data are available globally describing literal market exchange dynamics compared to total herd dynamics, a more refined analysis is not possible at global scale. Due to the lack of global data for live aquatic farmed animals (e.g., number and farm-gate prices), these could not be included in this part of the market value approximation.

Equation 1.1 uses the total number of head per livestock species reported for each year as the quantitative basis for analysis which values livestock at their average farm gate price, i.e., a point-in-time estimate (being the period of one year) of the total farmed animal stock. This assumption was required since the age structures of the herds for various farmed animal species in 181 countries were unavailable. This approximation approach may overvalue the market asset value for animals that have not reached slaughter age (e.g., calves) or passed their productive phase (e.g., dry cows) and may undervalue farmed animals that have a short life span (e.g., poultry). However, these population dynamics are assumed to be reflected in the average farm gate price per livestock species that was available.

Furthermore, the asset value is considered to capture producer and consumer (or purchaser) surplus from the market exchange of live animals, since the average farm gate price is assumed to represent a market situation where both are maximized.

***Equation 1.2 (asset value)***

When a dressing percentage was missing for a particular animal species and country (mostly affecting buffalo, camels, ducks, geese, rabbits, and horses for a very large number of countries) regional median values (e.g., from neighbouring countries) were imputed.

If carcass yield for terrestrial animal species were unavailable, records of the number of slaughtered animals and the associated output production volume were used to derive live weight conversion factors (Pelletier & Tyedmers, 2010). Carcass yields were then converted into live weight values for each animal species using dressing percentages provided via the technical conversion factors for agricultural commodities (FAO, 2021c).

***Equation 1.3 (asset value)***

An example for this calculation of the liveweight unit equivalent or biomass is provided below.

Table S.1: Data for calculating the asset value of farmed animals

| Country | Data | Data source |
| --- | --- | --- |
| Australia | Livestock type (Item) - Cattle (Heads): 27,412,872 | FAO (2021a), Data, Production ‘Crops and Livestock Products’ Table, Country: Australia Item: Live animals, Element: Stocks, Year: 2015 |
|  | Producer price (Item) - Cattle (USD/tonne): 1,185 | FAO (2021a), Data, Prices ‘Producer Prices’ Table, Country: Australia Item: Meat live weight, cattle, Element: Producer Price (USD/tonne), Months: Annual value, Year: 2015 |
|  | Producing Animals/Slaughtered – Cattle (Heads): 10103100  Production/Production Quantity -  Meat, cattle (tonnes): 2,661,640 | FAO (2021a), Data, Production ‘Crops and Livestock Products’ Table, Country: Australia Item: Livestock primary, Element: Producing Animals/Slaughtered, Year: 2015 |
|  | Conversion factors: Carcass weight as a % of live weight: 50% | FAO (2021c), Country: Australia, Livestock products: Cattle |

Equation 1.3:

$$\sum_{i,j} x_{i,j}f_{i.j}p_{i,j} \left( 1.3 \right)$$

Calculation:

Economic value of live cattle in Australian in 2015 = 27,412,872 (heads) x (2,661,640 (meat production quantity tonnes)/10,103,100 (Heads slaughtered)) x (1/0.5 (Carcass conversion)) x 1,185 (USD/tonne) = 17,112,704,685 ($ USD (US Dollars)).

This gives an average value of 624 USD per head of cattle.

Here, the average liveweight is calculated to be (2,661,640/10,103,100) / (1/0.5) = 527 kg, using a carcass percentage of 0.5.

***Equation 2 (output value)***

The market value of terrestrial and aquatic animal meat refers to production output in physical terms from slaughtered animals valued at the farm gate price of meat for each species within each country for a specific year (FAO, 2021d). Importantly, FAO (2021a) already provides the estimates for equation 2 for each output type, country, and year. These output market values are assumed to include added value such as transport, slaughter, and processing. Calculations of asset values assume that producer and consumer surplus are maximized and that costs incurred in generating a unit of output are reflected in average annual farm gate price (implying that producers aim for profit maximisation) of outputs in respective countries and in a specific year. The total global value for each output type and for each year was calculated by summing individual output market values for all countries.

***Equation 3 (total market value)***

Equation 3 is motivated by literature describing capital stock. Jarvis’ (1974) seminal study defines cattle as renewable capital stock in a portfolio. As such, the profile of stock value over time can be defined as V0, V1, V2, …, Vt. The change in value Δt can then be defined by linking the stock value in periods t+1 and t as Vt+1=Δt+ Vt. For physical capital stock that is non-renewable such as equipment Δt is governed by the difference between investment and depreciation (Usher, 1980). For biological capital stock that is renewable Δt is determined by investment, depreciation, and a stream of profit (value of production less input costs) from livestock production. For example, see Zhao, Wahl, and Marsh (2006) for a case study from the United States and Nogueira, Marsh, Tozer, and Peel (2011) for a case study from Mexico. In equation 3, input costs are unavailable and thus, not included, implying that Vt+1=Δt+ Vt ≤ VPt+ Vt where VPt is the value of production or revenue in t. Hence, equation 3 is an overestimate of total value, except where input costs are constant, so that there are no changes to them.

This approach includes the risk of double counting the present (potential) future output value as part of a farmed animal’s current asset market value. This is the market value of a live animal at a specific time (e.g., a steer at 12 months), which also includes a value describing some proportion of the animal’s future output (e.g., meat). The lack of global data describing herd age structure, salvage value, input costs, and depreciation rates (needed to derive salvage values) for the 181 countries currently prevents an analysis describing the scale of double counting between asset value (i.e., future output component) and output value.

Furthermore, there may also be an overlap in the number of live animals and animals that may have been slaughtered for meat (output) within a year. Unfortunately, the data source does not provide information about the potential extent of this overlap. This could be a further source of double counting of some of the economic value generated by farmed animals.

**Section 4: Additional Results**

Figure A.1: Global spatial distribution of the economic asset value by livestock type in 2018


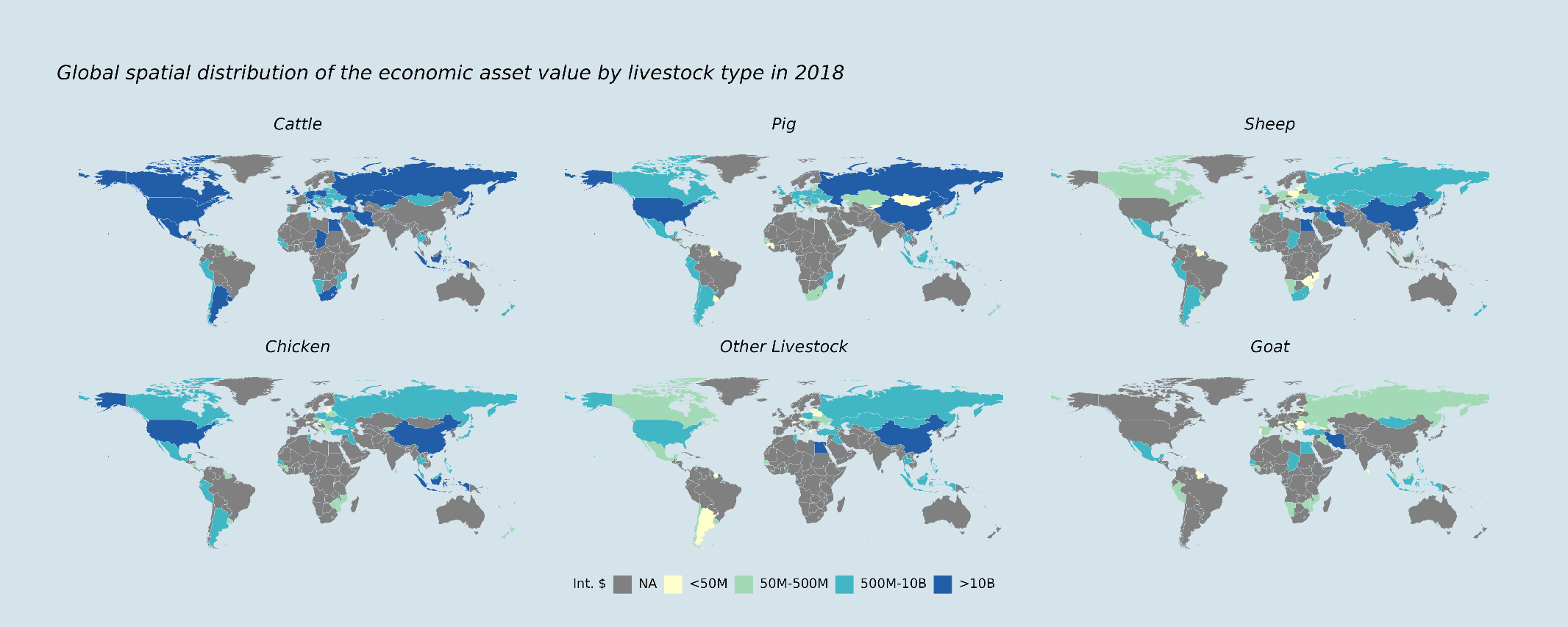


Notes: ‘Other’ includes horses, buffalo, asses, mules, and camels. ‘B’ describes for billion USD. Estimates exclude the stock value of live aquatic farmed animals due to a lack of global and price/value data. NA indicates that data was not available which includes either quantities or price/value data for 2018. Values are presented in International Dollar (2018). Source: FAO (2021a).

Figure A.2: Global spatial distribution of the economic value of direct farmed animal outputs (e.g., meat, milk, eggs, fish) in 2018


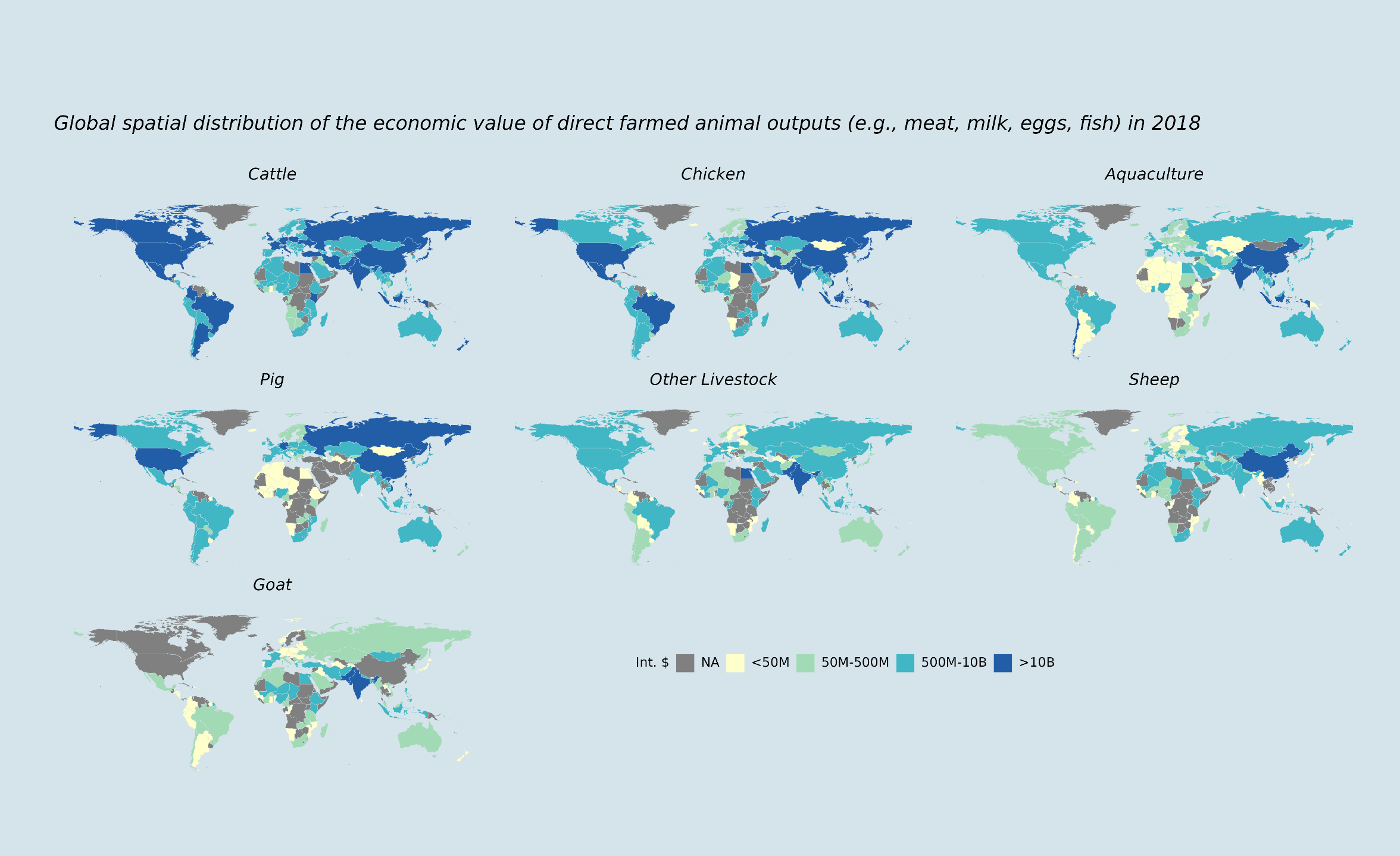


Notes: ‘B’ for billion USD, ‘M’ for million USD. NA indicates that data was not available which includes either quantities or price/value data for 2018. Values are presented in International Dollar (2018). Source: FAO (2021a), FAO (2021b).

Figure A.3: Absolute change in asset value between 2005-2018 by country income group


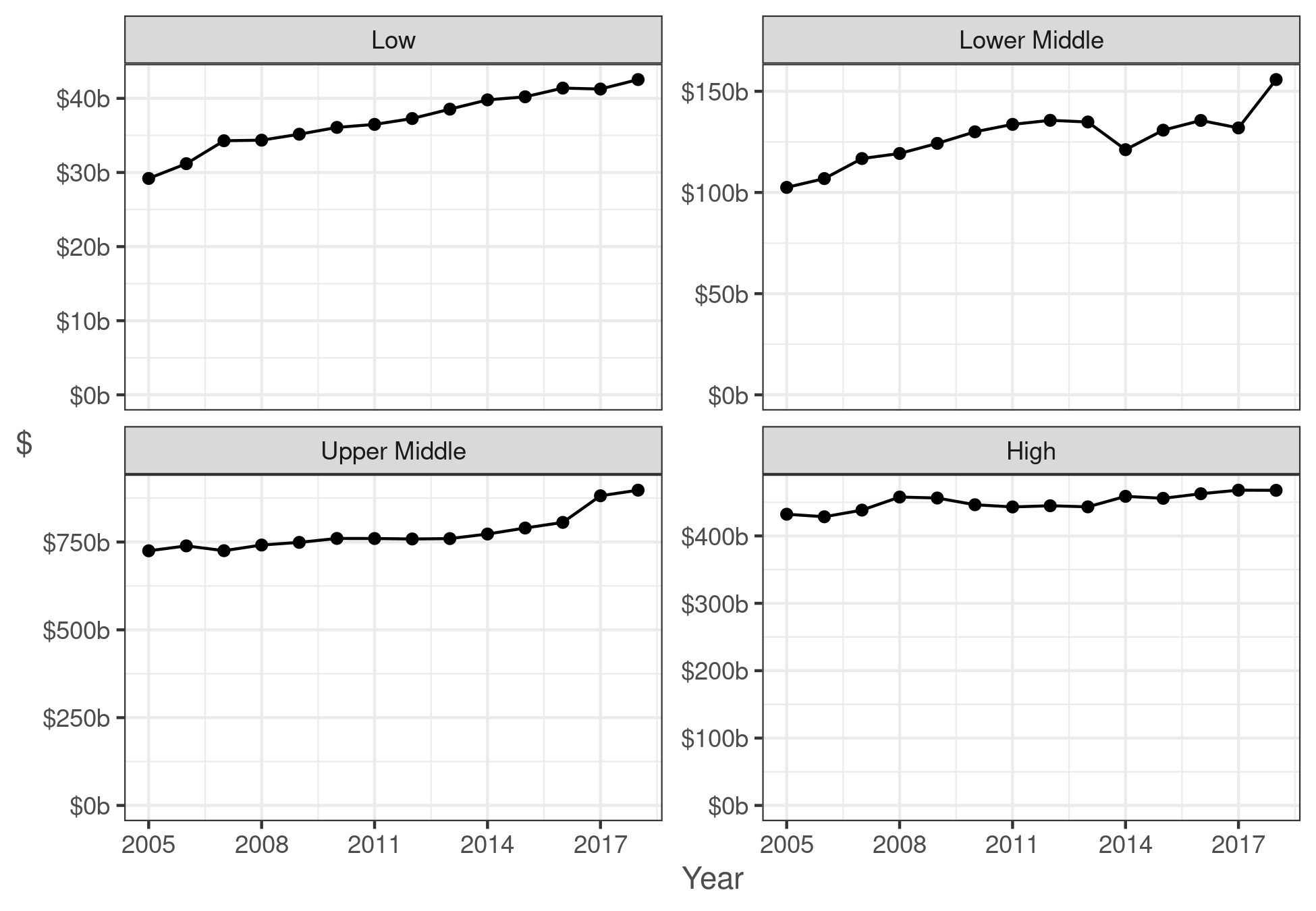


*Notes: All values in constant 2014-2016 USD. Sources: FAO (2021a), FAO (2021b), The World Bank (2021).*

Figure A.4: Absolute change in output value between 2005-2018 by country income group


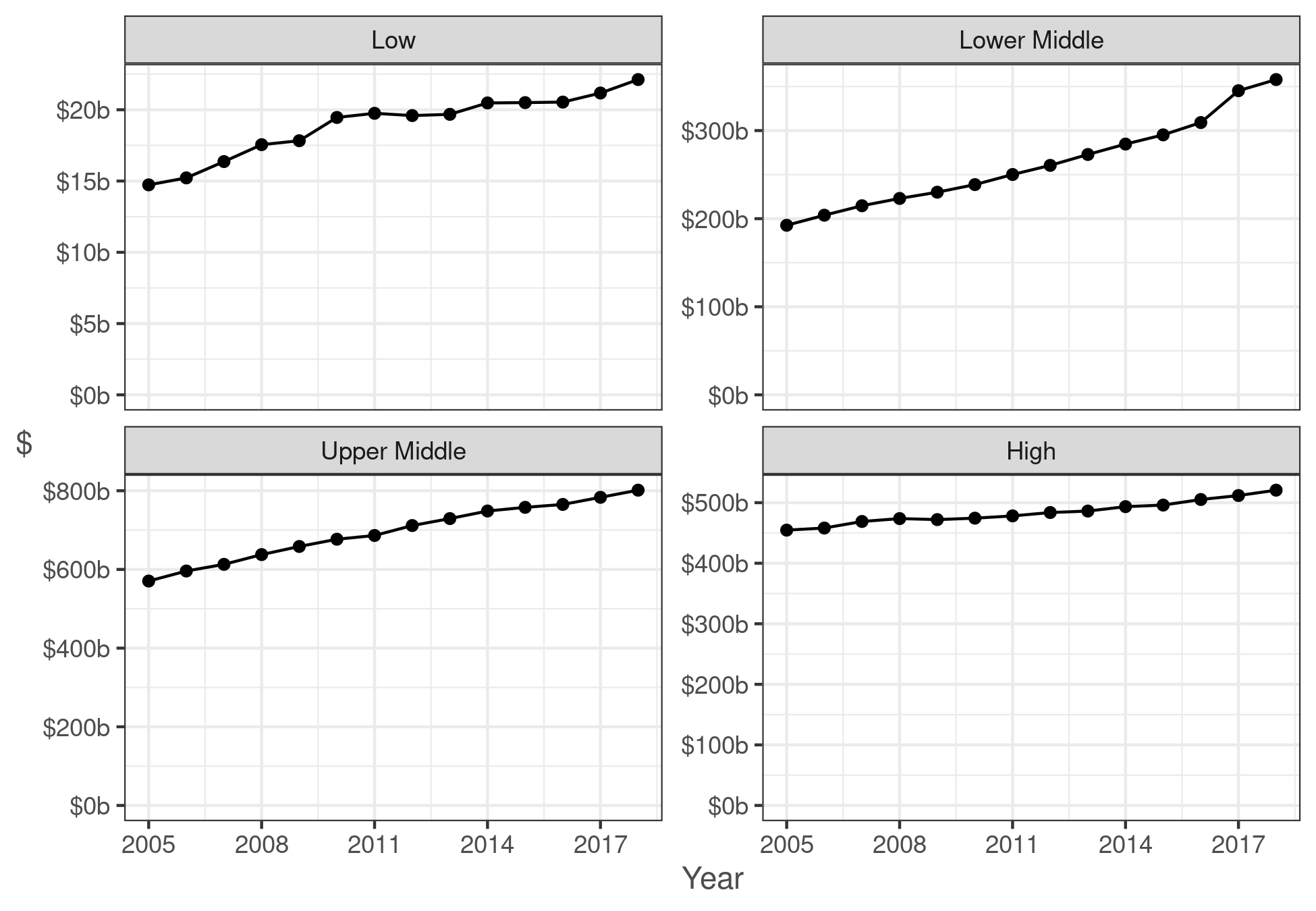


*Notes: All values in constant 2014-2016 USD. Sources: FAO (2021a), FAO (2021b), The World Bank (2021).*

Figure A.5: Per (human) capita change in asset value between 2005-2018 by country income group

*
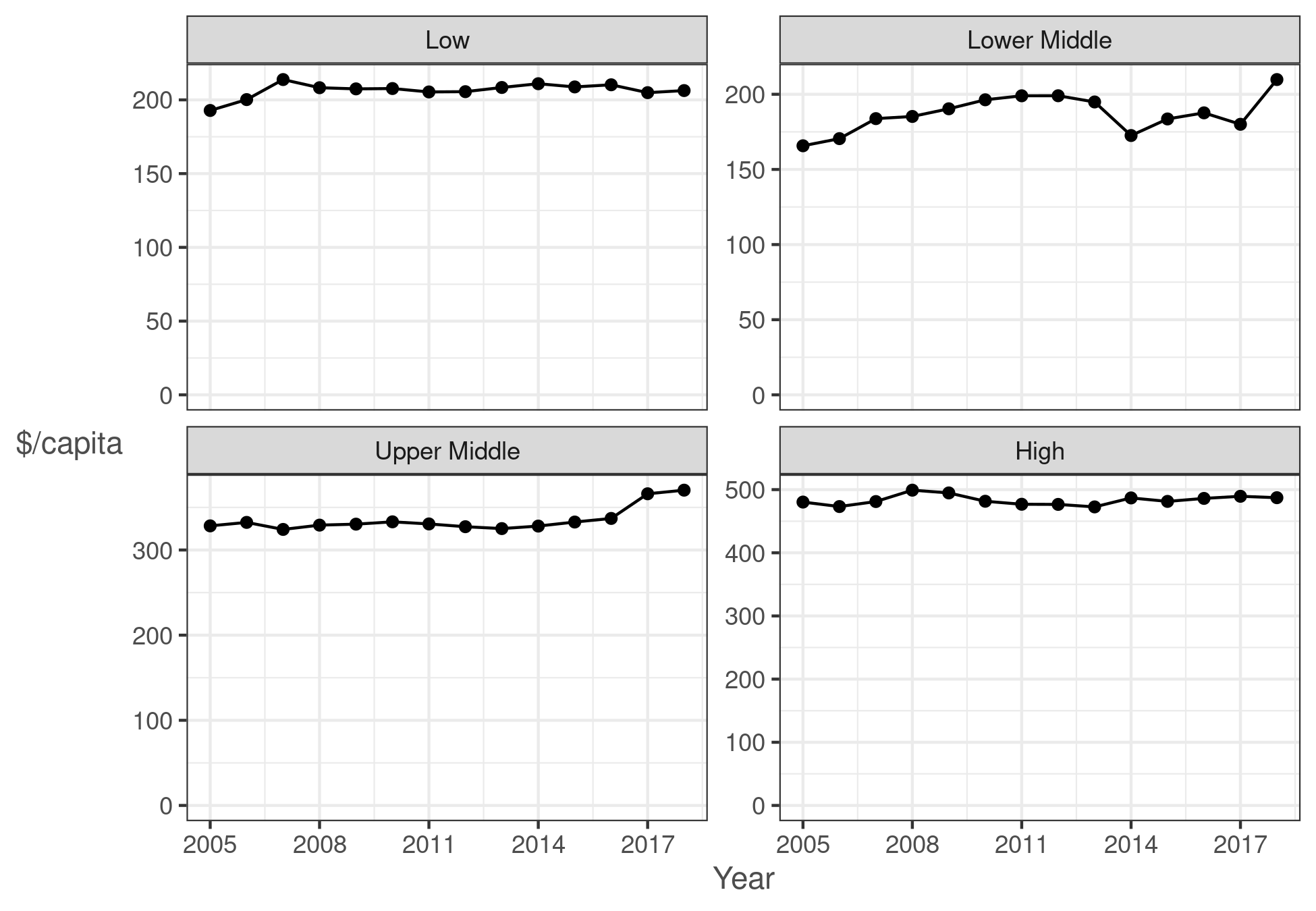
*

*Notes: All values in constant 2014-2016 USD. Sources: FAO (2021a), FAO (2021b), The World Bank (2021).*

Figure A.6: Per (human) capita change in output value between 2005-2018 by country income group


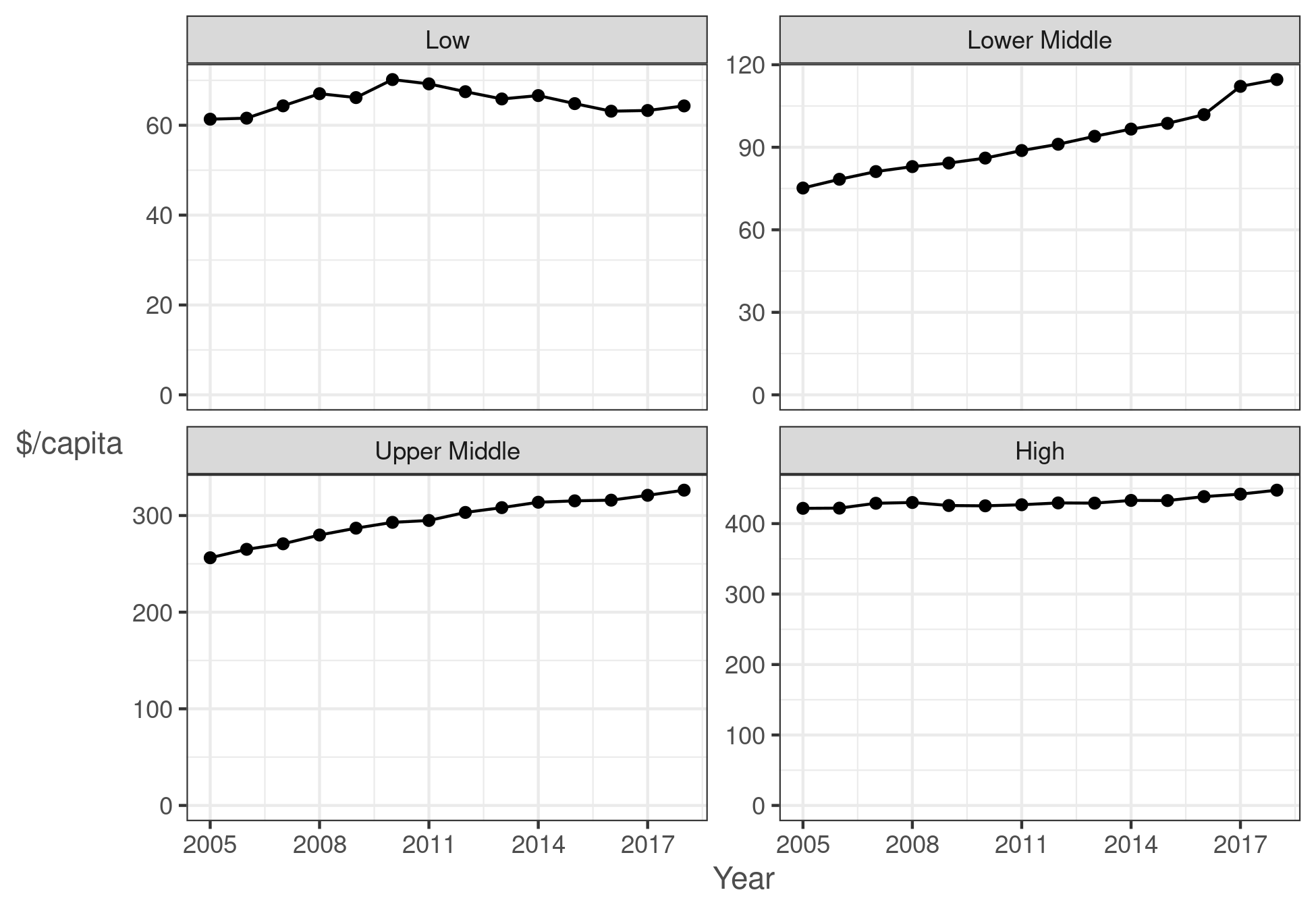


*Notes: All values in constant 2014-2016 USD. Sources: FAO (2021a), FAO (2021b), The World Bank (2021).*

Figure A.7: Absolute change in asset volume (tonnes) between 2005-2018 by country income group


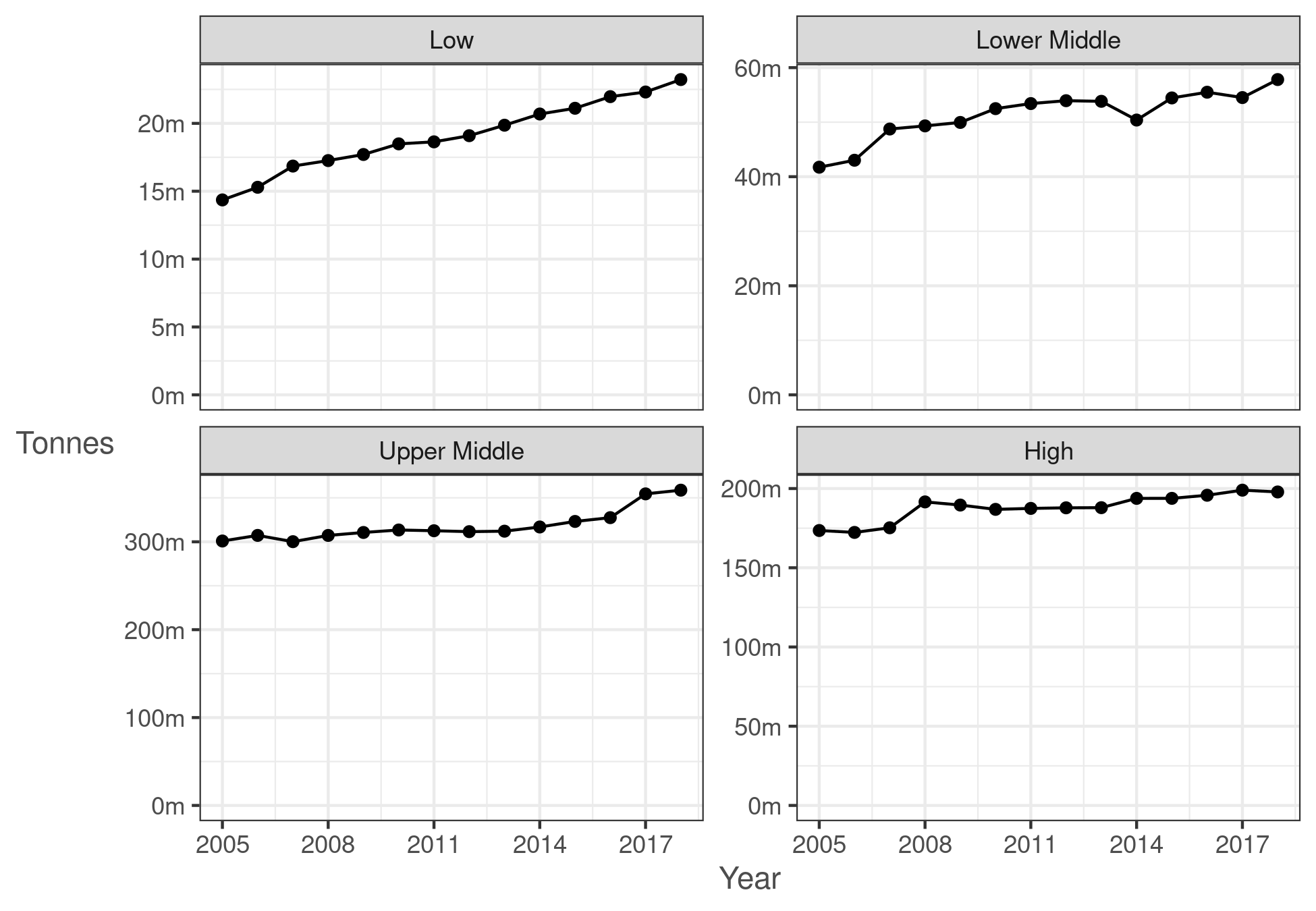


*Sources: FAO (2021a), FAO (2021b), The World Bank (2021).*

Figure A.8: Absolute change in output volume (tonnes) between 2005-2018 by country income group


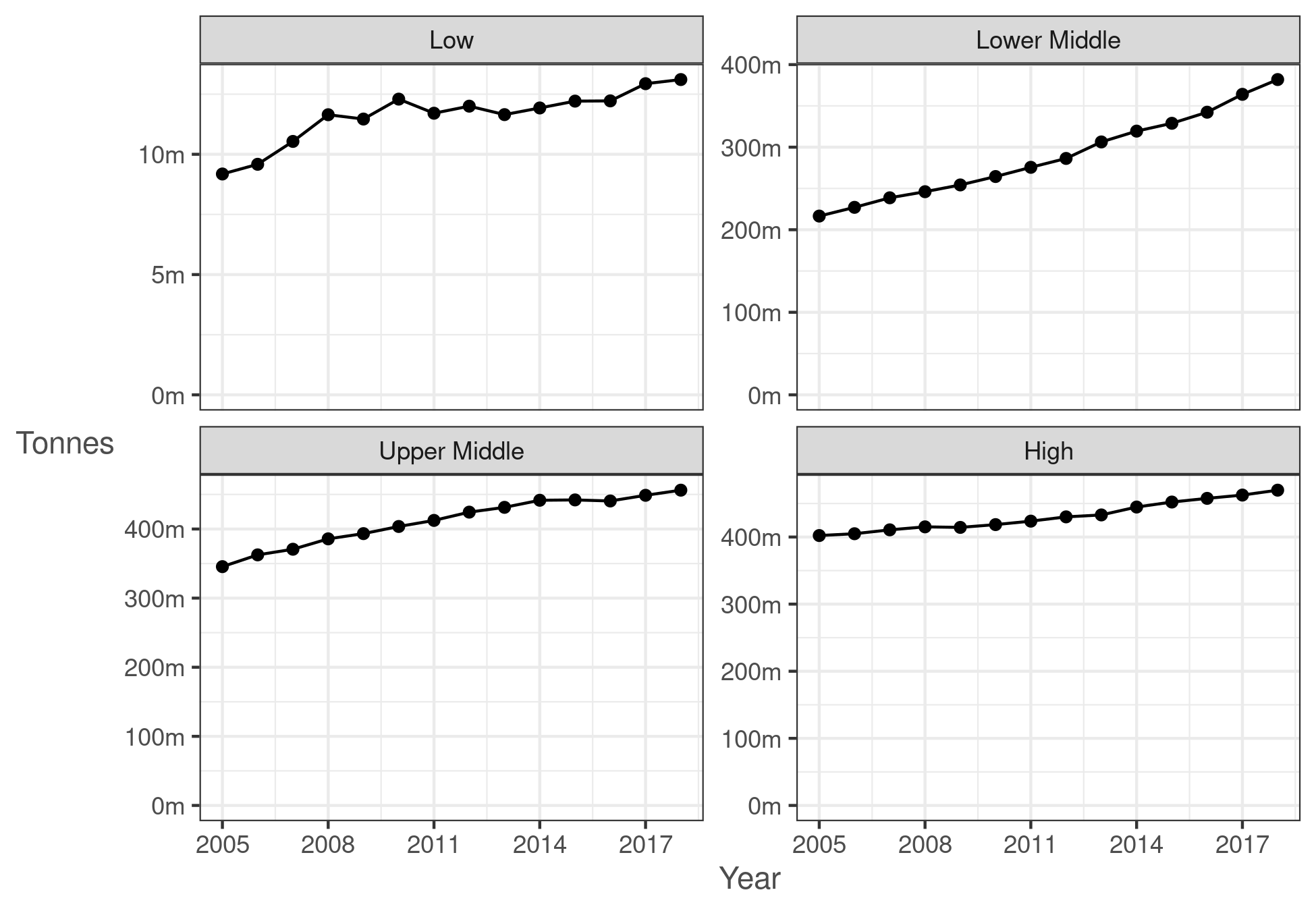


*Sources: FAO (2021a), FAO (2021b), The World Bank (2021).*

**Appendix: List of countries, livestock types, aquaculture species, outputs and crops included in the analysis**

***List of countries***

The analysis includes all countries listed in Table S.2. Data gaps for selected years may have resulted in not available (i.e., ‘N.A.’) observation for some countries within the analysis.

Table S.2: List of countries included in the analysis

| FAO country code | Country | FAO m49_code | FAO iso2_code | FAO iso3_code |
| --- | --- | --- | --- | --- |
| 2 | Afghanistan | 4 | AF | AFG |
| 3 | Albania | 8 | AL | ALB |
| 4 | Algeria | 12 | DZ | DZA |
| 5 | American Samoa | 16 | AS | ASM |
| 6 | Andorra | 20 | AD | AND |
| 7 | Angola | 24 | AO | AGO |
| 258 | Anguilla | 660 | AI | AIA |
| 8 | Antigua and Barbuda | 28 | AG | ATG |
| 9 | Argentina | 32 | AR | ARG |
| 1 | Armenia | 51 | AM | ARM |
| 22 | Aruba | 533 | AW | ABW |
| 10 | Australia | 36 | AU | AUS |
| 11 | Austria | 40 | AT | AUT |
| 52 | Azerbaijan | 31 | AZ | AZE |
| 12 | Bahamas | 44 | BS | BHS |
| 13 | Bahrain | 48 | BH | BHR |
| 16 | Bangladesh | 50 | BD | BGD |
| 14 | Barbados | 52 | BB | BRB |
| 57 | Belarus | 112 | BY | BLR |
| 255 | Belgium | 56 | BE | BEL |
| 15 | Belgium-Luxembourg | 58 | NA | NA |
| 23 | Belize | 84 | BZ | BLZ |
| 53 | Benin | 204 | BJ | BEN |
| 17 | Bermuda | 60 | BM | BMU |
| 18 | Bhutan | 64 | BT | BTN |
| 19 | Bolivia (Plurinational State of) | 68 | BO | BOL |
| 80 | Bosnia and Herzegovina | 70 | BA | BIH |
| 31 | Bouvet Island | 74 | BV | BVT |
| 21 | Brazil | 76 | BR | BRA |
| 26 | Brunei Darussalam | 96 | BN | BRN |
| 27 | Bulgaria | 100 | BG | BGR |
| 233 | Burkina Faso | 854 | BF | BFA |
| 29 | Burundi | 108 | BI | BDI |
| 35 | Cabo Verde | 132 | CV | CPV |
| 115 | Cambodia | 116 | KH | KHM |
| 32 | Cameroon | 120 | CM | CMR |
| 33 | Canada | 124 | CA | CAN |
| 37 | Central African Republic | 140 | CF | CAF |
| 39 | Chad | 148 | TD | TCD |
| 40 | Chile | 152 | CL | CHL |
| 96 | China, Hong Kong SAR | 344 | HK | HKG |
| 128 | China, Macao SAR | 446 | MO | MAC |
| 41 | China, mainland | 156 | CN | CHN |
| 214 | China, Taiwan Province of | 158 | TW | TWN |
| 44 | Colombia | 170 | CO | COL |
| 45 | Comoros | 174 | KM | COM |
| 46 | Congo | 178 | CG | COG |
| 48 | Costa Rica | 188 | CR | CRI |
| 107 | Côte d'Ivoire | 384 | CI | CIV |
| 98 | Croatia | 191 | HR | HRV |
| 49 | Cuba | 192 | CU | CUB |
| 279 | Curaçao | 531 | NA | CUW |
| 50 | Cyprus | 196 | CY | CYP |
| 167 | Czechia | 203 | CZ | CZE |
| 116 | Democratic People's Republic of Korea | 408 | KP | PRK |
| 250 | Democratic Republic of the Congo | 180 | CD | COD |
| 54 | Denmark | 208 | DK | DNK |
| 72 | Djibouti | 262 | DJ | DJI |
| 55 | Dominica | 212 | DM | DMA |
| 56 | Dominican Republic | 214 | DO | DOM |
| 58 | Ecuador | 218 | EC | ECU |
| 59 | Egypt | 818 | EG | EGY |
| 60 | El Salvador | 222 | SV | SLV |
| 61 | Equatorial Guinea | 226 | GQ | GNQ |
| 178 | Eritrea | 232 | ER | ERI |
| 63 | Estonia | 233 | EE | EST |
| 209 | Eswatini | 748 | SZ | SWZ |
| 238 | Ethiopia | 231 | ET | ETH |
| 64 | Faroe Islands | 234 | FO | FRO |
| 66 | Fiji | 242 | FJ | FJI |
| 67 | Finland | 246 | FI | FIN |
| 68 | France | 250 | FR | FRA |
| 69 | French Guyana | 254 | GF | GUF |
| 70 | French Polynesia | 258 | PF | PYF |
| 74 | Gabon | 266 | GA | GAB |
| 75 | Gambia | 270 | GM | GMB |
| 73 | Georgia | 268 | GE | GEO |
| 79 | Germany | 276 | DE | DEU |
| 81 | Ghana | 288 | GH | GHA |
| 84 | Greece | 300 | GR | GRC |
| 85 | Greenland | 304 | GL | GRL |
| 86 | Grenada | 308 | GD | GRD |
| 87 | Guadeloupe | 312 | GP | GLP |
| 88 | Guam | 316 | GU | GUM |
| 89 | Guatemala | 320 | GT | GTM |
| 90 | Guinea | 324 | GN | GIN |
| 175 | Guinea-Bissau | 624 | GW | GNB |
| 91 | Guyana | 328 | GY | GUY |
| 93 | Haiti | 332 | HT | HTI |
| 95 | Honduras | 340 | HN | HND |
| 97 | Hungary | 348 | HU | HUN |
| 99 | Iceland | 352 | IS | ISL |
| 100 | India | 356 | IN | IND |
| 101 | Indonesia | 360 | ID | IDN |
| 102 | Iran (Islamic Republic of) | 364 | IR | IRN |
| 103 | Iraq | 368 | IQ | IRQ |
| 104 | Ireland | 372 | IE | IRL |
| 105 | Israel | 376 | IL | ISR |
| 106 | Italy | 380 | IT | ITA |
| 109 | Jamaica | 388 | JM | JAM |
| 110 | Japan | 392 | JP | JPN |
| 112 | Jordan | 400 | JO | JOR |
| 108 | Kazakhstan | 398 | KZ | KAZ |
| 114 | Kenya | 404 | KE | KEN |
| 83 | Kiribati | 296 | KI | KIR |
| 118 | Kuwait | 414 | KW | KWT |
| 113 | Kyrgyzstan | 417 | KG | KGZ |
| 120 | Lao People's Democratic Republic | 418 | LA | LAO |
| 119 | Latvia | 428 | LV | LVA |
| 121 | Lebanon | 422 | LB | LBN |
| 122 | Lesotho | 426 | LS | LSO |
| 123 | Liberia | 430 | LR | LBR |
| 124 | Libya | 434 | LY | LBY |
| 125 | Liechtenstein | 438 | LI | LIE |
| 126 | Lithuania | 440 | LT | LTU |
| 256 | Luxembourg | 442 | LU | LUX |
| 129 | Madagascar | 450 | MG | MDG |
| 130 | Malawi | 454 | MW | MWI |
| 131 | Malaysia | 458 | MY | MYS |
| 132 | Maldives | 462 | MV | MDV |
| 133 | Mali | 466 | ML | MLI |
| 134 | Malta | 470 | MT | MLT |
| 127 | Marshall Islands | 584 | MH | MHL |
| 136 | Mauritania | 478 | MR | MRT |
| 137 | Mauritius | 480 | MU | MUS |
| 138 | Mexico | 484 | MX | MEX |
| 145 | Micronesia (Federated States of) | 583 | FM | FSM |
| 140 | Monaco | 492 | MC | MCO |
| 141 | Mongolia | 496 | MN | MNG |
| 273 | Montenegro | 499 | ME | MNE |
| 143 | Morocco | 504 | MA | MAR |
| 144 | Mozambique | 508 | MZ | MOZ |
| 28 | Myanmar | 104 | MM | MMR |
| 148 | Nauru | 520 | NR | NRU |
| 149 | Nepal | 524 | NP | NPL |
| 150 | Netherlands | 528 | NL | NLD |
| 153 | New Caledonia | 540 | NC | NCL |
| 156 | New Zealand | 554 | NZ | NZL |
| 157 | Nicaragua | 558 | NI | NIC |
| 158 | Niger | 562 | NE | NER |
| 159 | Nigeria | 566 | NG | NGA |
| 160 | Niue | 570 | NU | NIU |
| 154 | North Macedonia | 807 | MK | MKD |
| 162 | Norway | 578 | NO | NOR |
| 221 | Oman | 512 | OM | OMN |
| 165 | Pakistan | 586 | PK | PAK |
| 180 | Palau | 585 | PW | PLW |
| 166 | Panama | 591 | PA | PAN |
| 168 | Papua New Guinea | 598 | PG | PNG |
| 169 | Paraguay | 600 | PY | PRY |
| 170 | Peru | 604 | PE | PER |
| 171 | Philippines | 608 | PH | PHL |
| 173 | Poland | 616 | PL | POL |
| 174 | Portugal | 620 | PT | PRT |
| 179 | Qatar | 634 | QA | QAT |
| 117 | Republic of Korea | 410 | KR | KOR |
| 146 | Republic of Moldova | 498 | MD | MDA |
| 182 | Réunion | 638 | RE | REU |
| 183 | Romania | 642 | RO | ROU |
| 185 | Russian Federation | 643 | RU | RUS |
| 184 | Rwanda | 646 | RW | RWA |
| 188 | Saint Kitts and Nevis | 659 | KN | KNA |
| 189 | Saint Lucia | 662 | LC | LCA |
| 191 | Saint Vincent and the Grenadines | 670 | VC | VCT |
| 244 | Samoa | 882 | WS | WSM |
| 192 | San Marino | 674 | SM | SMR |
| 193 | Sao Tome and Principe | 678 | ST | STP |
| 194 | Saudi Arabia | 682 | SA | SAU |
| 195 | Senegal | 686 | SN | SEN |
| 272 | Serbia | 688 | RS | SRB |
| 186 | Serbia and Montenegro | 891 | CS | SCG |
| 197 | Sierra Leone | 694 | SL | SLE |
| 200 | Singapore | 702 | SG | SGP |
| 199 | Slovakia | 703 | SK | SVK |
| 198 | Slovenia | 705 | SI | SVN |
| 25 | Solomon Islands | 90 | SB | SLB |
| 201 | Somalia | 706 | SO | SOM |
| 202 | South Africa | 710 | ZA | ZAF |
| 277 | South Sudan | 728 | SS | SSD |
| 203 | Spain | 724 | ES | ESP |
| 38 | Sri Lanka | 144 | LK | LKA |
| 276 | Sudan | 729 | SD | SDN |
| 206 | Sudan (former) | 736 | NA | NA |
| 207 | Suriname | 740 | SR | SUR |
| 210 | Sweden | 752 | SE | SWE |
| 211 | Switzerland | 756 | CH | CHE |
| 212 | Syrian Arab Republic | 760 | SY | SYR |
| 208 | Tajikistan | 762 | TJ | TJK |
| 216 | Thailand | 764 | TH | THA |
| 176 | Timor-Leste | 626 | TL | TLS |
| 217 | Togo | 768 | TG | TGO |
| 218 | Tokelau | 772 | TK | TKL |
| 219 | Tonga | 776 | TO | TON |
| 220 | Trinidad and Tobago | 780 | TT | TTO |
| 222 | Tunisia | 788 | TN | TUN |
| 223 | Turkey | 792 | TR | TUR |
| 213 | Turkmenistan | 795 | TM | TKM |
| 227 | Tuvalu | 798 | TV | TUV |
| 226 | Uganda | 800 | UG | UGA |
| 230 | Ukraine | 804 | UA | UKR |
| 225 | United Arab Emirates | 784 | AE | ARE |
| 229 | United Kingdom of Great Britain and Northern Ireland | 826 | GB | GBR |
| 215 | United Republic of Tanzania | 834 | TZ | TZA |
| 231 | United States of America | 840 | US | USA |
| 234 | Uruguay | 858 | UY | URY |
| 235 | Uzbekistan | 860 | UZ | UZB |
| 155 | Vanuatu | 548 | VU | VUT |
| 236 | Venezuela (Bolivarian Republic of) | 862 | VE | VEN |
| 237 | Viet Nam | 704 | VN | VNM |
| 249 | Yemen | 887 | YE | YEM |
| 251 | Zambia | 894 | ZM | ZMB |
| 181 | Zimbabwe | 716 | ZW | ZWE |

Source: FAO (2021b).

***Aquaculture species***

Output data for global aquaculture production was sourced through FAO (2021b). For the analysis the aquaculture species listed within FAO (2021b) in file ‘CL_FI_SPECIES_GROUPS’ under category Yearbook Group "Fish, crustaceans and molluscs, etc." (i.e., approximately 630 listed species). Excluded from the "Fish, crustaceans and molluscs, etc." were pearls, mother-of-pearl, shells.

***Outputs***

Data about outputs from framed animal production and crops was obtained from FAO (2021a) for terrestrial farmed animals and FAO (2021b) for aquatic farmed animals. The list of outputs includes meat (terrestrial animals), eggs, milk, fish (i.e., different forms such as whole fish, fillet, in shell). While quantities for offal, fat and other secondary outputs were available for livestock species in FAO (2021a), price data for these outputs were missing.

***Crop types***

The list crops included in the analysis was derived from FAO (2021a) is shown in Table S.2.

Table S.2: List of crop types included in the analysis

| Item (as listed in FAOSTAT) | FAO item code | FAO group |
| --- | --- | --- |
| Almonds_with_shell | 221 | fruits_nuts |
| Anise_badian_fennel_coriander | 711 | coffe_spice_crops |
| Apples | 515 | fruits_nuts |
| Apricots | 526 | fruits_nuts |
| Areca_nuts | 226 | fruits_nuts |
| Artichokes | 366 | vegetables |
| Asparagus | 367 | vegetables |
| Avocados | 572 | fruits_nuts |
| Bambara_beans | 203 | pulses |
| Bananas | 486 | fruits_nuts |
| Barley | 44 | cereals |
| Beans_dry | 176 | pulses |
| Beans_green | 414 | vegetables |
| Berries_nes | 558 | fruits_nuts |
| Blueberries | 552 | fruits_nuts |
| Brazil_nuts_with_shell | 216 | fruits_nuts |
| Broad_beans_horse_beans_dry | 181 | pulses |
| Buckwheat | 89 | cereals |
| Cabbages_and_other_brassicas | 358 | vegetables |
| Canary_seed | 101 | cereals |
| Carobs | 461 | fruits_nuts |
| Carrots_and_turnips | 426 | vegetables |
| Cashew_nuts_with_shell | 217 | fruits_nuts |
| Cashewapple | 591 | fruits_nuts |
| Cassava | 125 | roots_tubers |
| Cassava_leaves | 378 | vegetables |
| Castor_oil_seed | 265 | oilseed_oil_fruits |
| Cauliflowers_and_broccoli | 393 | vegetables |
| Cereals_nes | 108 | cereals |
| Cherries | 531 | fruits_nuts |
| Cherries_sour | 530 | fruits_nuts |
| Chestnut | 220 | fruits_nuts |
| Chick_peas | 191 | pulses |
| Chicory_roots | 459 | coffe_spice_crops |
| Chillies_and_peppers_dry | 689 | coffe_spice_crops |
| Chillies_and_peppers_green | 401 | vegetables |
| Cinnamon_cannella | 693 | coffe_spice_crops |
| Cloves | 698 | coffe_spice_crops |
| Cocoa_beans | 661 | coffe_spice_crops |
| Coconuts | 249 | oilseed_oil_fruits |
| Coffee_green | 656 | coffe_spice_crops |
| Cottonseed | 329 | oilseed_oil_fruits |
| Cow_peas_dry | 195 | pulses |
| Cranberries | 554 | fruits_nuts |
| Cucumbers_and_gherkins | 397 | vegetables |
| Currants | 550 | fruits_nuts |
| Cates | 577 | fruits_nuts |
| Eggplants_aubergines | 399 | vegetables |
| Figs | 569 | fruits_nuts |
| Fonio | 94 | cereals |
| Fruit_citrus_nes | 512 | fruits_nuts |
| Fruit_fresh_nes | 619 | fruits_nuts |
| Fruit_pome_nes | 542 | fruits_nuts |
| Fruit_stone_nes | 541 | fruits_nuts |
| Fruit_tropical_fresh_nes | 603 | fruits_nuts |
| Garlic | 406 | vegetables |
| Ginger | 720 | coffe_spice_crops |
| Gooseberries | 549 | fruits_nuts |
| Grain_mixed | 103 | cereals |
| Grapefruit_inc_pomelos | 507 | fruits_nuts |
| Grapes | 560 | fruits_nuts |
| Groundnuts_with_shell | 242 | oilseed_oil_fruits |
| Hazelnuts_with_shell | 225 | fruits_nuts |
| Hempseed | 336 | oilseed_oil_fruits |
| Hops | 677 | coffe_spice_crops |
| Jojoba_seed | 277 | oilseed_oil_fruits |
| Kapok_fruit | 310 | oilseed_oil_fruits |
| Kapokseed_in_shell | 311 | oilseed_oil_fruits |
| Karite_nuts_sheanuts | 263 | oilseed_oil_fruits |
| Kiwi_fruit | 592 | fruits_nuts |
| Kola_nuts | 224 | fruits_nuts |
| Leeks_other_alliaceous_vegetables | 407 | vegetables |
| Lemons_and_limes | 497 | fruits_nuts |
| Lentils | 201 | pulses |
| Lettuce_and_chicory | 372 | vegetables |
| Linseed | 333 | oilseed_oil_fruits |
| Lupins | 210 | pulses |
| Maize | 56 | cereals |
| Maize_green | 446 | vegetables |
| Mangoes_mangosteens_guavas | 571 | fruits_nuts |
| Melons_other_inc_cantaloupes | 568 | vegetables |
| Melonseed | 299 | oilseed_oil_fruits |
| Millet | 79 | cereals |
| Mushrooms_and_truffles | 449 | vegetables |
| Mustard_seed | 292 | oilseed_oil_fruits |
| Nutmeg_mace_and_cardamoms | 702 | coffe_spice_crops |
| Nuts_nes | 234 | fruits_nuts |
| Oats | 75 | cereals |
| Oil_palm_fruit | 254 | oilseed_oil_fruits |
| Oilseeds_nes | 339 | oilseed_oil_fruits |
| Okra | 430 | vegetables |
| Olives | 260 | oilseed_oil_fruits |
| Onions_dry | 403 | vegetables |
| Onions_shallots_green | 402 | vegetables |
| Oranges | 490 | fruits_nuts |
| Palm_kernels | 256 | oilseed_oil_fruits |
| Papayas | 600 | fruits_nuts |
| Peaches_and_nectarines | 534 | fruits_nuts |
| Pears | 521 | fruits_nuts |
| Peas_dry | 187 | pulses |
| Peas_green | 417 | vegetables |
| Pepper_piper_spp | 687 | coffe_spice_crops |
| Persimmons | 587 | fruits_nuts |
| Pigeon_peas | 197 | pulses |
| Pineapples | 574 | fruits_nuts |
| Pistachios | 223 | fruits_nuts |
| Plantains_and_others | 489 | fruits_nuts |
| Plums_and_sloes | 536 | fruits_nuts |
| Poppy_seed | 296 | oilseed_oil_fruits |
| Potatoes | 116 | roots_tubers |
| Pulses_nes | 211 | pulses |
| Pumpkins_squash_and_gourds | 394 | vegetables |
| Quinces | 523 | fruits_nuts |
| Quinoa | 92 | cereals |
| Rapeseed | 270 | oilseed_oil_fruits |
| Raspberries | 547 | fruits_nuts |
| Rice_paddy | 27 | cereals |
| Roots_and_tubers_nes | 149 | roots_tubers |
| Rye | 71 | cereals |
| Safflower_seed | 280 | oilseed_oil_fruits |
| Sesame_seed | 289 | oilseed_oil_fruits |
| Sorghum | 83 | cereals |
| Soybeans | 236 | oilseed_oil_fruits |
| Spices_nes | 723 | coffe_spice_crops |
| Spinach | 373 | vegetables |
| Strawberries | 544 | fruits_nuts |
| String_beans | 423 | vegetables |
| Sugar_beet | 157 | sugar_crops |
| Sugar_cane | 156 | sugar_crops |
| Sugar_crops_nes | 161 | sugar_crops |
| Sunflower_seed | 267 | oilseed_oil_fruits |
| Sweet_potatoes | 122 | roots_tubers |
| Tallowtree_seed | 305 | oilseed_oil_fruits |
| Tangerines_mandarins_clementines_satsumas | 495 | fruits_nuts |
| Taro_cocoyam | 136 | roots_tubers |
| Tea | 667 | coffe_spice_crops |
| Tomatoes | 388 | vegetables |
| Triticale | 97 | cereals |
| Tung_nuts | 275 | oilseed_oil_fruits |
| Vanilla | 692 | coffe_spice_crops |
| Vegetables_fresh_nes | 463 | vegetables |
| Vegetables_leguminous_nes | 420 | vegetables |
| Vetches | 205 | pulses |
| Walnuts_with_shell | 222 | fruits_nuts |
| Watermelons | 567 | vegetables |
| Wheat | 15 | cereals |
| Yams | 137 | roots_tubers |
| Yautia_cocoyam | 135 | roots_tubers |

Source: FAO (2021a)

**References**

Barrett, C. B., Chabari, F., Bailey, D., Little, P. D., & Coppock, D. L. (2003). Livestock Pricing in the Northern Kenyan Rangelands. *Journal of African Economies, 12*(2), 127-155. doi:<https://doi.org/10.1093/jae/12.2.127>

Bateman, I. J., Carson, R. T., Day, B., Hanemann, M., Hanley, N., Hett, T., . . . Loomes, G. (2002). *Economic Valuation with Stated Preference Techniques: A Manual*. Cheltenham, UK: ElgarOnline. Edward Elgar Publishing.

Bilmes, L. J., & Loomis, J. B. (2019). *Valuing U.S. National Parks and Programs: America’s Best Investment* London. UK: Routledge.

Conte, M. N. (2013). Valuing Ecosystem Services. In S. A. Levin (Ed.), *Encyclopedia of Biodiversity (Second Edition)* (pp. 314-326). Waltham: Academic Press.

Deloitte Access Economics. (2017). *Total economic value of the Great Barrier Reef*. Brisbane, Australia: Deloitte Access Economics Pty Ltd. Retrieved from <https://www.barrierreef.org/uploads/Total%20economic%20value%20of%20the%20Great%20Barrier%20Reef%20-%20Need%20to%20know.pdf>

Drucker, A. G., Gomez, V., & Anderson, S. (2001). The economic valuation of farm animal genetic resources: a survey of available methods. *Ecological Economics, 36*(1), 1-18. doi:<https://doi.org/10.1016/S0921-8009(00)00242-1>

Ejlertsen, M., Poole, J., & Marshall, K. (2012). Traditional breeding objectives and practices of goat, sheep and cattle smallholders in The Gambia and implications in relation to the design of breeding interventions. *Tropical Animal Health and Production, 45*(1), 219-229. doi:<https://doi.org/10.1007/s11250-012-0194-1>

Emerton, L. (2018). Economic Valuation of Wetlands: Total Economic Value. In C. M. Finlayson, M. Everard, K. Irvine, R. J. McInnes, B. A. Middleton, A. A. van Dam, & N. C. Davidson (Eds.), *The Wetland Book: I: Structure and Function, Management, and Methods* (pp. 2127-2132). Dordrecht: Springer Netherlands.

FAO. (2021a). *FAOSTAT*. Rome, Italy: Food and Agriculture Organization of the United Nations (FAO). Retrieved from <https://www.fao.org/faostat/en/#data>

FAO. (2021b). *FishStatJ - Software for Fishery and Aquaculture Statistical Time Series*. Rome, Italy: Food and Agriculture Organization of the United Nations (FAO) Retrieved from <https://www.fao.org/fishery/statistics/software/fishstatj/en>

FAO. (2021c). *Technical conversion factors for agricultural commodities*. Rome, Italy: Food and Agriculture Organization of the United Nations (FAO) Retrieved from <https://www.fao.org/fileadmin/templates/ess/documents/methodology/tcf.pdf>

FAO. (2021d). *Value of Agriculture Production (Related Documents)*. Rome, Italy: Food and Agriculture Organization of the United Nations (FAO) Retrieved from <https://fenixservices.fao.org/faostat/static/documents/QV/QV_e.pdf>

Hensher, D. A., Rose, J. M., & Greene, W. H. (2015). *Applied Choice Analysis* (2 ed.). Cambridge: Cambridge University Press.

Jarvis, L. S. (1974). Cattle as capital goods and ranchers as portfolio managers: An application to the Argentine cattle sector. *Journal of Political Economy, 82*(3), 489-520. doi:<https://www.jstor.org/stable/1829842>

National Research Council. (1999). *Perspectives on Biodiversity: Valuing Its Role in an Everchanging World*. Washington, DC: The National Academies Press.

Nogueira, L., Marsh, T. L., Tozer, P. R., & Peel, D. (2011). Foot-and-mouth disease and the Mexican cattle industry. *42*(s1), 33-44. doi:<https://doi.org/10.1111/j.1574-0862.2011.00550.x>

Nyariki, D. M., & Amwata, D. A. (2019). The value of pastoralism in Kenya: Application of total economic value approach. *Pastoralism, 9*(1), 9. doi:<https://doi.org/10.1186/s13570-019-0144-x>

Pearce, D., Atkinson, G., & Mourato, S. (2006). *Cost-Benefit Analysis and the Environment - Recent Developments*. Paris, France: Oaganisation for Economic Co-operation and Development (OECD). Retrieved from <https://www.oecd-ilibrary.org/docserver/9789264010055-en.pdf?expires=1627880202&id=id&accname=ocid177482a&checksum=69CFB7341E4422468CA7A90414661CCA>

Pelletier, N., & Tyedmers, P. (2010). Forecasting potential global environmental costs of livestock production 2000–2050. *Proceedings of the National Academy of Sciences, 107*(43), 18371-18374. doi:<https://doi.org/10.1073/pnas.1004659107>

Robinson, T. P., Thornton, P. K., Franceschini, G., Kruska, R. L., Chiozza, F., Notenbaert, A., . . . See, L. (2011). *Global livestock production systems*. Rome, Italy: Food and Agriculture Organization of the United Nations (FAO) and International Livestock Research Institute (ILRI) Retrieved from <https://cgspace.cgiar.org/bitstream/handle/10568/10537/faoglobalLivestock.pdf>

The World Bank. (2021). *Population, total*. Washington, USA: The World Bank Retrieved from <https://data.worldbank.org/indicator/SP.POP.TOTL>

Usher, D. (1980). *The Measurement of Capital*. In D. Usher (Ed.), *National Bureau of Economic Research Books*.

Whitehead, J. C., & Haab, T. C. (2013). Contingent Valuation Method. In J. F. Shogren (Ed.), *Encyclopedia of Energy, Natural Resource, and Environmental Economics* (pp. 334-341). Waltham: Elsevier.

Zander, K. K., & Drucker, A. G. (2008). Conserving what's important: Using choice model scenarios to value local cattle breeds in East Africa. *Ecological Economics, 68*(1), 34-45. doi:<https://doi.org/10.1016/j.ecolecon.2008.01.023>

Zander, K. K., Signorello, G., De Salvo, M., Gandini, G., & Drucker, A. G. (2013). Assessing the total economic value of threatened livestock breeds in Italy: Implications for conservation policy. *Ecological Economics, 93*, 219-229. doi:<https://doi.org/10.1016/j.ecolecon.2013.06.002>

Zhao, Z., Wahl, T. I., & Marsh, T. L. (2006). Invasive Species Management: Foot-and-Mouth Disease in the U.S. Beef Industry. *Agricultural and Resource Economics Review, 35*(1), 98-115. doi:<https://doi.org/10.1017/S106828050001008X>
